# KAP1 in antiviral immunity: dual roles in viral silencing and immune regulation

**DOI:** 10.3389/fcimb.2025.1618103

**Published:** 2025-10-02

**Authors:** Ruihua Xin, Mutien-Marie Garigliany, Jianxi Li

**Affiliations:** ^1^ Key Laboratory of Veterinary Pharmaceutical Development of Ministry of Agriculture, Technology Innovation Center of Traditional Chinese Veterinary Medicine of Gansu Province, Lanzhou Institute of Husbandry and Pharmaceutical Sciences, Chinese Academy of Agricultural Sciences, Lanzhou, China; ^2^ Fundamental and Applied Research for Animals & Health (FARAH), INDEEP, Laboratory of Pathology, Faculty of Veterinary Medicine, University of Liège, Liège, Belgium

**Keywords:** KAP1/TRIM28, transcriptional regulation, virus–host interaction, innate immunity, post-translational modifications, viral latency

## Abstract

Krüppel-associated box (KRAB)-associated protein 1 (KAP1), also known as TRIM28 due to its tripartite motif (TRIM) domain, is a member of the transcription intermediary factor 1 (TIF1) family. Since its discovery in 1996, KAP1 has been widely studied as a scaffold protein involved in histone methylation, heterochromatin formation, and genome maintenance. Its function and stability are dynamically regulated by post-translational modifications (PTMs), including phosphorylation, SUMOylation, and acetylation. In addition, KAP1 serves as a signal transducer via its SUMO/ubiquitin E3 ligase activity. This review summarizes current advances in understanding the roles of KAP1 in regulating retroviruses (RVs), herpesviruses, and emerging respiratory viruses such as Severe Acute Respiratory Syndrome Coronavirus 2 (SARS-CoV-2) and influenza A virus (IAV), with a particular focus on the interplay between its structural domains and physiological functions. Recent findings on human immunodeficiency virus (HIV) are highlighted to address ongoing mechanistic controversies, particularly those involving KAP1-mediated latency control. We further examine novel insights into KAP1’s involvement in other viruses, including hepatitis B virus (HBV), porcine reproductive and respiratory syndrome virus (PRRSV), and African swine fever virus (ASFV). as well as its emerging regulatory roles in host innate immune responses through PTM-mediated modulation of antiviral signaling pathways. Although KAP1 exerts both antiviral and proviral effects, the underlying mechanisms remain incompletely defined, especially in systems where conflicting observations exist for the same pathogen. These discrepancies—reflecting both methodological variation and KAP1’s inherent regulatory complexity—underscore the need for deeper mechanistic insight. Future studies utilizing precise genetic tools and *in vivo* models will be critical for elucidating the context-specific roles of KAP1 in viral gene regulation and advancing its translational potential.

## 1 Introduction

Krüppel-associated box (KRAB)-associated protein 1 (KAP1) is a transcriptional cofactor first identified and cloned by Friedman’s team in 1996 using affinity chromatography, and named for its ability to bind KRAB domain-containing zinc finger proteins (KRAB-ZFPs) (Friedman et al., 1996). The same year, another group independently identified the protein and named it KRIP1 (KRAB-A interacting protein 1) (Kim et al., 1996). Subsequent studies have shown that KAP1 can also interact with proteins lacking the KRAB domain, such as MDM2 and c-Myc, thereby modulating the transcription of their target genes (Wang et al., 2005; Kimura et al., 2007). The N-terminal RBCC (RING-B box-coiled coil) domain of KAP1 contains a zinc finger, two B-boxes, and a coiled-coil region, forming the tripartite motif (TRIM) structure. KAP1 is structurally related to other TRIM family E3 ligases, including TRIM24 (TIF1α), TRIM33 (TIF1γ), and TRIM66 (TIF1δ), which together comprise the transcription intermediary factor 1 (TIF1) family (Zhu and Xiao, 2024). Accordingly, KAP1 is also referred to as tripartite motif-containing protein 28 (TRIM28) or TIF1β (Cheng, 2014).

Accumulating evidence suggests that members of the TIF1 family contribute to genome stability through chromatin-based regulation of transcription and DNA damage response (McAvera and Crawford, 2020; Kotobuki et al., 2021). KAP1 is predominantly localized in the nucleus and possesses evolutionarily conserved structural domains (Iyengar and Farnham, 2011; Rosspopoff and Trono, 2023). The RBCC domain is crucial for multimerization and interaction with KRAB domains, facilitating protein-protein interactions, while the C-terminal PHD (plant homeodomain) and bromodomain (BrD) are primarily involved in chromatin modification and transcriptional regulation (Padeken et al., 2022; Grewal, 2023). Due to its modular domain architecture, KAP1 plays multiple physiological roles (Cheng, 2014), with most studies focusing on its transcriptional regulatory functions. For instance, KAP1 acts as a transcriptional co-repressor by recruiting histone methyltransferases to KRAB-ZFP target sites, thereby promoting heterochromatin formation and gene silencing (Kim et al., 1996; Cheng, 2014; Santoni De Sio, 2014). It also mediates chromatin remodeling in response to DNA damage, contributing to genomic stability and DNA repair. Beyond transcriptional regulation, KAP1 also functions as a SUMO/E3 ubiquitin ligase and a signaling scaffold, participating in diverse signaling pathways (Cheng, 2014; Bürck et al., 2016). KAP1 is subject to various post-translational modifications (PTMs), including serine phosphorylation, SUMOylation, and acetylation. These modifications regulate its function and protein abundance, allowing KAP1 to coordinate diverse cellular processes such as DNA repair, cytokine production, and stem cell maintenance. Notably, KAP1 knockout results in embryonic lethality, highlighting its essential role in development (Cammas et al., 2000). Conditional KAP1 deficiency leads to impaired erythropoiesis, abnormal T and B lymphocyte differentiation, and defective spermatogenesis, further underscoring its physiological significance (Cheng, 2014).

In disease contexts, KAP1 is closely associated with tumor development and progression. Elevated KAP1 expression is correlated with poor prognosis in cervical, gastric, ovarian, and hepatocellular carcinomas, and it has been proposed as a biomarker to distinguish glioblastoma from lower-grade gliomas (Jovčevska et al., 2017). Conversely, in early-stage lung cancer, high KAP1 expression is associated with improved overall survival (Czerwińska et al., 2017; Park et al., 2021), suggesting potential anti-proliferative roles. Given these findings, the complex relationship between KAP1 and cancer has been extensively reviewed elsewhere (Czerwińska et al., 2017) and will not be discussed in detail here.

KAP1 also plays multifaceted roles in viral infection (Randolph et al., 2022). Early studies focused on its function in silencing endogenous retroviruses (ERVs) through heterochromatin formation to maintain genomic stability (Sun et al., 2019; Geis and Goff, 2020; Asimi et al., 2022; Taka et al., 2022). KAP1 has since been implicated in the regulation of exogenous retroviruses (RVs) such as human immunodeficiency virus (HIV) (Yuan et al., 2021; Randolph et al., 2022), though whether it promotes latency or activation of HIV-1 remains controversial. In addition to RVs, KAP1 exerts dual regulatory roles on herpesviruses, including Epstein-Barr virus (EBV) (Xu et al., 2023) and Kaposi’s sarcoma-associated herpesvirus (KSHV) (Li et al., 2018). KAP1 maintains herpesvirus latency by silencing viral genes; however, host kinases and inflammasomes hijacked by the virus can induce phosphorylation of KAP1 at Ser824, disrupting latency and facilitating viral reactivation and replication (Li et al., 2019; Burton et al., 2020; Bhaduri-McIntosh and Rousseau, 2024).

Recent studies have linked KAP1 to disease severity in Severe Acute Respiratory Syndrome Coronavirus 2 (SARS-CoV-2) infection and host immune modulation (Ren et al., 2024). Moreover, KAP1 has been shown to influence the replication and immune evasion of several DNA and RNA viruses, including influenza A virus (IAV), African swine fever virus (ASFV), and human papillomavirus (HPV). These effects are mediated through the impact of KAP1 on viral replication, degradation of viral proteins, autophagy, immune escape, and coagulation regulation (Chang et al., 2021; Köcher et al., 2022; Zhu et al., 2024). Importantly, KAP1 regulates host antiviral innate immunity by modulating key signaling proteins such as RIG-I, MAVS, and TBK1 via its SUMOylation and ubiquitin ligase activities (Li et al., 2021; Chen et al., 2023).

In this review, we summarize the current knowledge regarding KAP1’s structural features and core cellular functions, with a particular focus on its roles in viral infection and antiviral innate immunity.

## 2 KAP1 protein structure and functional modules

**Figure 1:**
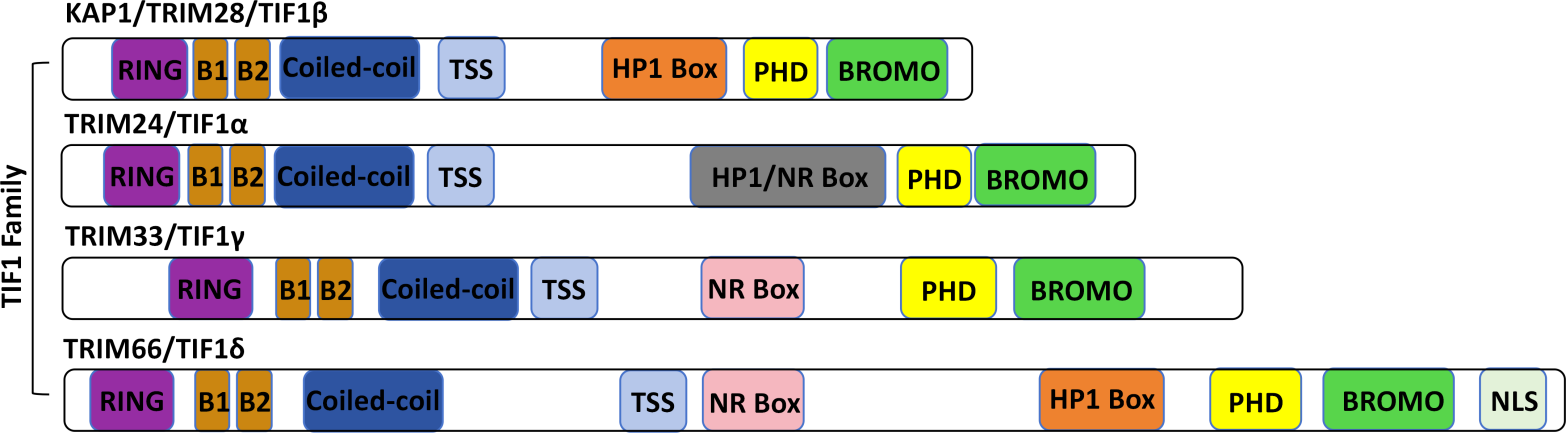
Protein structure of the TIF1 protein family. The TIF1 family includes TRIM24 (TIF1α), TRIM28 (TIF1β/KAP1), TRIM33 (TIF1γ), and TRIM66 (TIF1δ). All members share a characteristic N-terminal RBCC motif-comprising a RING finger, B-boxes, and a coiled-coil domain-and a C-terminal tandem plant homeodomain (PHD) and bromodomain. Variations in the central region distinguish each family member.

**Figure 2:**
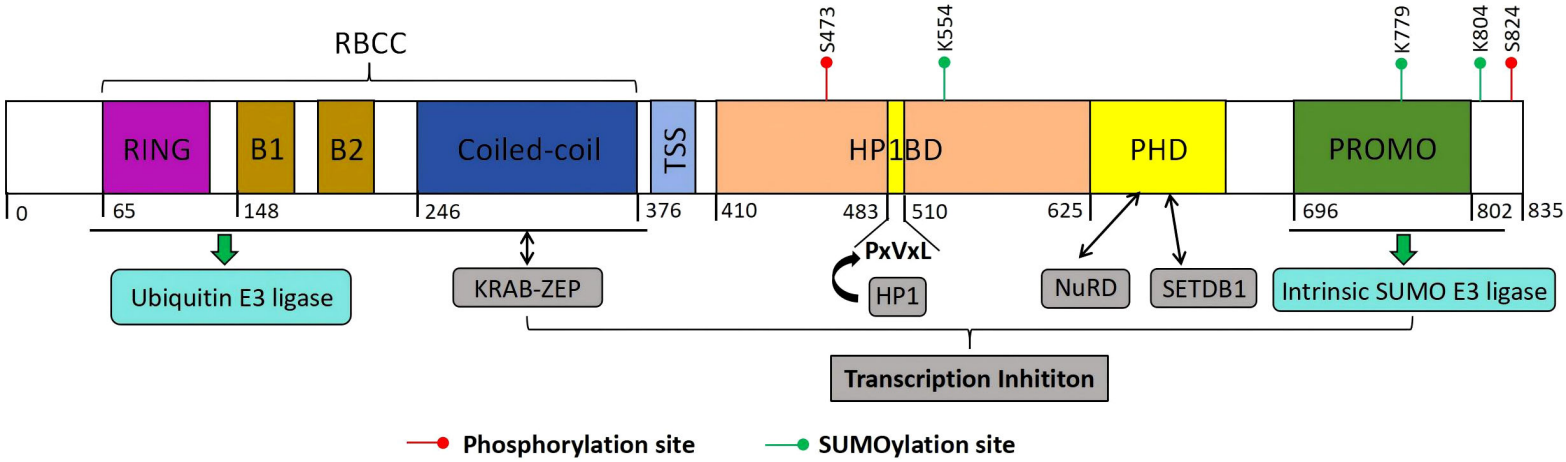
Structure of KAP1. KAP1 is composed of an N-terminal RING domain, two B-boxes (B1 and B2), a coiled-coil (CC) domain, a central hydrophobic PxVxL pentapeptide motif, and a C-terminal plant homeodomain (PHD) followed by a bromodomain. The RBCC region functions both as a ubiquitin E3 ligase and as a binding interface for KRAB-ZFPs. The PHD domain facilitates the recruitment of the NuRD complex and the histone methyltransferase SETDB1, while the central PxVxL motif mediates interaction with heterochromatin protein 1 (HP1). KAP1’s transcriptional repression activity is tightly associated with its ability to recruit SETDB1, NuRD, and HP1, and is further modulated by SUMOylation. Notably, the PHD domain itself possesses E3 SUMO ligase activity. Post-translational modifications of KAP1, including SUMOylation and ubiquitination at distinct sites, exert differential effects on its function and regulatory capacity.

All members of the TIF1 family share a conserved modular architecture (Khetchoumian et al., 2004): the N-terminal RBCC domain comprises a RING finger, two B-box zinc finger motifs, and a coiled-coil region. The central region, known as the TIF1 signature sequence (TSS), is the least conserved and is enriched in proline, glycine, and serine residues. The C-terminal region includes a conserved plant homeodomain (PHD) followed by a bromodomain (Friedman et al., 1996; Venturini et al., 1999). KAP1, along with TIF1α and TIF1δ, possesses a central heterochromatin protein 1 (HP1) binding domain (**Figure 1**). Unlike other TIF1 family members, however, KAP1 lacks a nuclear receptor (NR) box (Peng et al., 2000). Structurally, KAP1 is highly modular, with discrete domains that facilitate oligomerization and SUMOylation, enable recruitment to target genes by transcription factors, serve as scaffolds for chromatin-modifying enzymes, and mediate interaction with HP1 (Venturini et al., 1999). Each domain of KAP1 contributes distinctly to its roles in transcriptional repression and post-translational modifications (**Figure 2**).

### 2.1 RBCC structural domains: mediators of protein interactions and post-translational modifications

The RBCC region (tripartite motif, TRIM) of KAP1 is composed of a RING zinc finger, two B-box motifs, and a coiled-coil (CC) domain, arranged sequentially from the N– to the C-terminus (Friedman et al., 1996; Venturini et al., 1999; Meroni and Diez-Roux, 2005). This region mediates protein-protein interactions and drives KAP1’s dimerization and oligomerization. The RING domain adopts a C3HC4-type zinc finger fold and coordinates two zinc ions to form a hydrophobic core, with conserved linkers contributing to substrate specificity (Zeng et al., 2008). It also functions as an E3 ubiquitin ligase by recruiting E2 conjugating enzymes to mediate substrate ubiquitination (Metzger et al., 2014). The B-box motifs form compact globular structures conserved across TRIM proteins (Peng et al., 2000). The CC domain consists of amphipathic α-helices that mediate homo–and hetero-oligomerization with TRIM proteins (Stoll et al., 2019; Sun et al., 2019).

This structural module also enables direct interaction with KRAB-ZFPs, the largest family of transcriptional repressors in mammals (Ecco et al., 2017). The KRAB domain recruits KAP1 to DNA-binding loci to mediate silencing (Ecco et al., 2017; Sun et al., 2019). Among TIF1 family proteins, only KAP1 binds directly and specifically to the KRAB repression module (Stoll et al., 2022). Early work proposed a 3:1 KAP1:KRAB stoichiometry (Peng et al., 2000, 2007), whereas recent studies demonstrate a 1:2 KRAB-ZFP: KAP1 binding ratio. KAP1’s RBCC forms antiparallel dimers that can assemble into higher-order oligomers, though these are not required for repression (Fonti et al., 2019; Stoll et al., 2019). AlphaFold2 shows L301 inserts into a KRAB hydrophobic pocket, with CC helices forming the interaction site. Structural variations here may tune silencing efficacy (Taka et al., 2022).

In addition to mediating protein interactions, KAP1’s RBCC and adjacent regions undergo extensive post-translational modifications. Key lysines in the C-terminal region are SUMOylated, primarily by PIAS family ligases that recognize ψKxE motifs (Gareau and Lima, 2010; Zhao, 2018). Lysine 554 (K554), near the HP1-binding domain (residues 535-580), is the primary SUMO1 site and promotes HP1 interaction and heterochromatin formation (Lee et al., 2007). K779 (SUMO2/3) is linked to DNA repair (Zheng et al., 2000), while K804 SUMOylation may affect H3K9me3 binding (Lee et al., 2007). These modifications recruit SIM–containing partners like SETDB1, promoting H3K9 trimethylation and transcriptional silencing (Liu et al., 2021b). KAP1’s RING domain also mediates E3 ubiquitin ligase activity, requiring Cys15, Cys18, His30, and Cys33 for zinc coordination and E2 enzyme recruitment (Kim et al., 1996). Trp22 and Phe25 contribute to a hydrophobic pocket essential for ubiquitin transfer (Nyenhuis et al., 2025). B-box (residues 96-140) and CC (residues 141-350) domains enhance dimerization and substrate recognition, including of p53 and MDM2 (Peng et al., 2000).

SUMOylation and ubiquitination are spatially and functionally interlinked. SUMOylation may mask the RING domain and inhibit ubiquitin ligase activity (Cheng, 2014). Upon DNA damage, SUMOylated KAP1 can promote ubiquitination of repair factors, targeting them for degradation (Kuo et al., 2014; Shah et al., 2022). These PTMs dynamically regulate KAP1’s transcriptional repression and DNA repair roles via conformational modulation and signaling crosstalk (Cheng, 2014).

### 2.2 PHD-bromodomain: mediators of histone methylation and deacetylation

The C-terminal region of KAP1 contains two highly conserved domains: a plant homeodomain (PHD) zinc finger and a bromodomain. The PHD is a compact domain of approximately 60 amino acids, while the bromodomain comprises over 100 amino acids that fold into a helical bundle. Tandemly arranged, the PHD-Bromo module recognizes histone tails, recruits histone modifiers, and initiates SUMOylation, thereby promoting gene silencing (Peng and Wysocka, 2008; Zeng et al., 2008). Proteins interacting with the PHD-Bromo module fall into two main categories. The first includes histone methyltransferases like SETDB1, which catalyzes H3K9me2/3—a heterochromatic mark that recruits downstream effectors (Padeken et al., 2022; Grewal, 2023). The second group includes HDAC complexes (e.g., Mi2α, HDAC1), which assemble into larger repressive structures like NCOR2 and the NuRD complex. They remove histone acetyl groups, promoting chromatin compaction and repression (Sahu et al., 2024). Thus, the PHD-Bromo domain acts as a scaffold for histone modifiers, facilitating methylation and heterochromatin formation essential for gene silencing (Fukuda and Shinkai, 2020).

### 2.3 HP1-binding domain: recruitment of heterochromatin protein 1

The interaction between HP1 and members of the TIF1 protein family was first identified through yeast two-hybrid screening (Venturini et al., 1999). HP1 is a highly conserved non-histone protein that plays a key role in heterochromatin formation (Sales-Gil and Vagnarelli, 2020; Schoelz and Riddle, 2022). HP1 consists of an N-terminal chromodomain (CD) and a C-terminal chromoshadow domain (CSD), both essential for recognizing histone marks and mediating chromatin compaction (Sales-Gil and Vagnarelli, 2020). KAP1 contains a central HP1-binding domain (HP1BD), defined by a conserved PxVxL motif, although it is the least conserved domain within KAP1 (Thiru et al., 2004). Following deposition of H3K9me3, KAP1 rapidly binds HP1 with high affinity. This occurs through interaction between KAP1’s PxVxL motif and the HP1 CSD, facilitating heterochromatin assembly (Lechner et al., 2000; Sripathy et al., 2006). The KAP1-HP1 interaction is essential for transcriptional silencing. Disrupting this interaction, such as by altering histone or DNA methylation, can lead to *in vivo* reactivation of imprinted genes (Iyengar et al., 2011). Moreover, HP1 has been shown to associate with the histone lysine methyltransferase SUV39H1 and to form a multiprotein complex with KAP1. This complex acts as both a “writer” and “reader” of H3K9me2/3, reinforcing and maintaining heterochromatin stability (Wang et al., 2019).

## 3 Core physiological functions of KAP1

### 3.1 Maintaining a heterochromatin environment and mediating gene silencing

Heterochromatin is a condensed chromatin state that represses transcription *in vivo*. It is organized around nucleosomes composed of histone octamers (H3, H4, H2A, H2B) wrapped by DNA (Sahu et al., 2024). Heterochromatin can be categorized into two types: constitutive and facultative. Constitutive heterochromatin contains abundant non-histone proteins like HP1 and KAP1, which help maintain genome stability in eukaryotic cells (Grewal, 2023). Key PTMs include H3K9me3, H3K64me3, and H4K20me3—hallmarks of constitutive heterochromatin (Wang et al., 2019; Ballmer et al., 2023). Among known histone lysine methyltransferases (KMTs), SETDB1 plays a dominant role in catalyzing H3K9 methylation (Fukuda and Shinkai, 2020; Lin et al., 2021; Rapone et al., 2023). Chromatin remodelers like CHD3 and HDAC1 also interact with KAP1 to facilitate nucleosome remodeling (Goodarzi et al., 2011).

In canonical gene silencing, KRAB-ZFPs and other DNA-binding proteins recruit KAP1 to specific genomic loci (Ecco et al., 2017; Stoll et al., 2022; Taka et al., 2022). KAP1 then recruits the NuRD-HDAC complex and SETDB1 to deposit H3K9me3, establishing heterochromatin and repressing transcription (Stoll et al., 2022). Simultaneously, the PHD-bromodomain module of KAP1 recruits the SUMO E2 enzyme UBC9, facilitating auto-SUMOylation of KAP1. This modification enhances SETDB1 recruitment and stabilizes chromatin repression (Xu et al., 2023).

KAP1 also regulates transcriptional elongation to reinforce gene silencing (McNamara et al., 2016b). After transcription is initiated, RNA polymerase II (RNAP II) typically pauses just downstream of the transcription start site (TSS). Productive elongation requires activation of positive transcription elongation factor b (P-TEFb), composed of CDK9 and cyclin T (Yang et al., 2021). P-TEFb phosphorylates the C-terminal domain (CTD) of Pol II and the negative elongation factors such as NELF and DSIF to facilitate elongation. Under resting conditions, P-TEFb is sequestered in the 7SK small nuclear ribonucleoprotein (7SK snRNP) complex, which includes 7SK RNA, HEXIM1/2, and LARP7 (Schneeberger et al., 2019), thereby maintaining Pol II in a paused state (D’Orso, 2016; McNamara et al., 2016b).

Importantly, KAP1 was found to recruit the 7SK snRNP complex, containing inactive P-TEFb, to paused promoters, including the HIV-1 LTR. While this recruitment enforces pausing under basal conditions, it also primes these promoters for rapid reactivation, since upon stimulation P-TEFb can be released and activated, relieving Pol II pausing and favoring HIV-1 transcription (McNamara et al., 2016b). KAP1 promotes SUMOylation of CDK9 at lysine residues (e.g., K44, K56, K68), which destabilizes the P-TEFb complex (Ma et al., 2019). KAP1 also enhances the association of P-TEFb with 7SK snRNP via HEXIM1, reinforcing transcriptional pausing under basal conditions (McNamara et al., 2016a). Phosphorylation of KAP1 at Ser824 disrupts its interaction with SETDB1 and HP1, leading to the release of P-TEFb from the 7SK snRNP complex. This promotes the transition of Pol II into productive elongation, enabling rapid transcription of target genes (McNamara et al., 2016a; Yang et al., 2021). Additionally, Bacon et al. (2020) reported that the PHD domain of KAP1 binds hypoacetylated histone H4, potentially influencing Pol II recruitment or termination (Bunch and Calderwood, 2015).

Recent studies have uncovered additional mechanisms by which KAP1 regulates gene expression. Citrullination of KAP1, catalyzed by peptidylarginine deiminase 4 (PADI4), enhances its interaction with the chromatin remodeler Smarcad1, loosens chromatin compaction, and promotes the transcription of pluripotency genes such as *Nanog* and *Klf4*. This is achieved through increased H3K27ac and H3K4me3, along with decreased H3K9me3 at regulatory regions, thereby weakening KAP1 repressive function. Thus, KAP1 citrullination serves as a critical epigenetic mechanism for activating pluripotency genes in embryonic stem cells (ESCs) (Zhang et al., 2023b). KAP1 also facilitates transcriptional repression by Kaiso (ZBTB33), a zinc finger and BTB domain-containing repressor, through its PHD-bromodomain, which interacts with both the BTB/POZ and zinc finger regions of Kaiso and promotes its SUMOylation (Lobanova et al., 2023). Furthermore, KAP1 maintains H3K9me3 levels and the heterochromatic environment through continuous SETDB1 recruitment, forming a complex with chromatin assembly factor 1 (CAF-1) and HP1 (Lechner et al., 2000). Although phosphorylation at KAP1 Ser473 has been implicated in maintaining global H3K9me3 (Chang et al., 2008; Bolderson et al., 2012), the precise role of this modification remains unclear.

**Figure 3:**
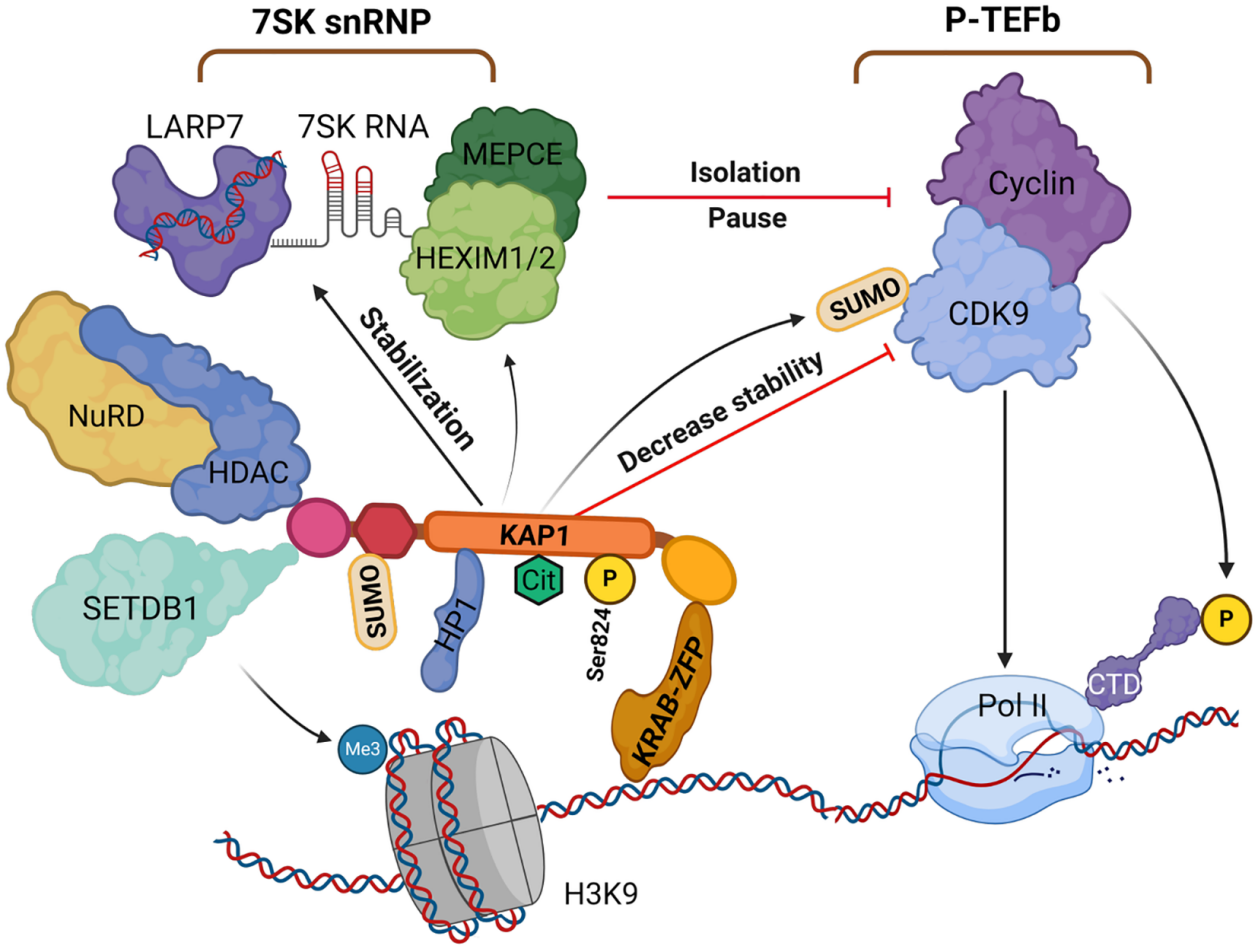
The role of KAP1 in gene silencing and transcriptional repression. KAP1 is recruited by KRAB-ZFPs to specific DNA loci, where it initiates heterochromatin formation via SETDB1 and the NuRD-HDAC complex, depositing H3K9me3 and promoting histone deacetylation. HP1 binds H3K9me3 and stabilizes the complex. KAP1 undergoes SUMOylation, enhancing its repressive activity, and further SUMOylates CDK9 to inhibit P-TEFb assembly and stabilize the 7SK snRNP complex, pausing RNAPII at promoters. Citrullination and phosphorylation (Ser824) dynamically modulate KAP1’s function and chromatin interactions. Created with BioRender.com. H3K9, histone H3 lysine 9; HP1, heterochromatin protein 1; KAP1, KRAB-associated protein 1; NuRD-HDAC, nucleosome remodeling and deacetylase complex; P-TEFb, positive transcription elongation factor b; Pol II, RNAPII; SETDB1, SET domain bifurcated histone methyltransferase 1; snRNP, small nuclear ribonucleoprotein; KRAB-ZFP, KRAB zinc finger protein.

In summary, KAP1 is targeted to specific genomic loci, such as transposable elements or imprinted genes, through its interaction with KRAB-ZFPs. It then recruits SETDB1 to deposit H3K9me3, together with HDACs and HP1, thereby establishing and maintaining heterochromatin and enforcing gene silencing. In parallel, KAP1 suppresses transcriptional elongation by promoting SUMOylation of CDK9 and enhancing the sequestration of P-TEFb in the 7SK snRNP complex, thereby maintaining RNAPII in a paused state (Schultz et al., 2002; Sripathy et al., 2006; Sun et al., 2019). Auto-SUMOylation of KAP1 is essential for maintaining repression, whereas Ser824 phosphorylation reverses this state by displacing CHD3 and promoting chromatin relaxation (**Figure 3**) (Goodarzi et al., 2011). This dynamic regulation enables KAP1 to coordinate chromatin remodeling and transcriptional control in response to endogenous cues and external stimuli, including pathogen invasion.

### 3.2 Involvement in DNA damage repair

DNA damage arises from both exogenous sources–such as ultraviolet (UV) and ionizing radiation–and endogenous events, including replication stress and enzymatic errors (Carusillo and Mussolino, 2020). To maintain genomic stability, cells have evolved a highly coordinated DNA damage response (DDR) system (Oksenych and Kainov, 2021) that rapidly detects lesions, initiates repair pathways, and temporarily halts cell cycle progression to prevent propagation of mutations (Huang and Zhou, 2020). DDR encompasses multiple repair mechanisms, including direct repair, base excision repair, nucleotide excision repair, mismatch repair, and double-strand break (DSB) repair (Da Costa and Schmidt, 2020). Among these, DSBs are particularly harmful and are recognized by two sensor complexes: the MRE11-RAD50-NBS1 (MRN) complex and the Ku70/Ku80 heterodimer, which activate homologous recombination (HR) and non-homologous end joining (NHEJ), respectively (Tan et al., 2023; Liu et al., 2024).

Phosphorylation of KAP1 is among the earliest DDR events and is primarily linked to DSB repair within heterochromatin (Ziegler et al., 2020). In microlaser-irradiated U2OS human osteosarcoma cells, KAP1 rapidly localizes to damage sites, where it is recognized by the MRN complex and recruits Ataxia Telangiectasia Mutated (ATM) kinase. ATM phosphorylates KAP1 at Ser824 (Bhatia et al., 2013), leading to chromatin relaxation–a prerequisite for DSB repair. Phosphorylated KAP1 has been shown to co-localize with key DNA repair factors, including γH2AX, 53BP1, and TopBP1, underscoring its involvement in HR-mediated repair (White et al., 2006).

KAP1 deacetylation enhances its interaction with 53BP1 and facilitates ATM-independent NHEJ (Lin et al., 2015). DNA damage also triggers Ser473 phosphorylation of KAP1, promoting formation of a KAP1–PCNA–Suv39h1 complex. Unlike Ser824, which localizes to damage foci, Ser473 phosphorylation is diffusely nuclear and mediated by Chk1/Chk2 (Hu et al., 2012). It helps maintain global H3K9me3 levels and contributes to H4K20me3 and H3K64me3 deposition (Hu et al., 2012; White et al., 2012). Thus, under normal physiological conditions, Ser473 phosphorylation promotes heterochromatin stability and replication fidelity (White et al., 2012). Upon DSBs, KAP1 undergoes Ser824 phosphorylation or deacetylation via ATM-dependent signaling to enable DNA repair (White et al., 2012).

KAP1-mediated DDR is further modulated by regulatory proteins. RNF4 recruitment to KAP1 modulates the 53BP1–BRCA1 balance at DSBs and influences pathway choice in a cell cycle–dependent manner (Kuo et al., 2016). While Ser824 phosphorylation is essential for DSB repair in heterochromatin, PP4C impairs this process by dephosphorylating Ser824. PP4C thus serves as a negative regulator of the DDR (Lee et al., 2012).

KAP1 also integrates external stimuli into DDR responses. Viral mimics such as poly I:C or direct infection can induce Ser473 phosphorylation. This promotes KAP1–CTIF interaction, which inhibits stress granule formation and restricts viral replication (Chang et al., 2021).

### 3.3 SUMOylation and ubiquitination in KAP1-mediated regulation

KAP1 acts as both a SUMO and E3 ubiquitin ligase, with these PTMs critically contributing to genomic stability, transcriptional regulation, and cell fate decisions. By coordinating protein interactions, chromatin remodeling, and intracellular signaling, KAP1 serves as a central regulatory hub (Cheng, 2014). SUMOylation enables KAP1 to recruit effector proteins containing SUMO-interacting motifs (SIMs), including HP1α/β/γ and SETDB1. This modification strengthens KAP1–HP1 binding and promotes HP1 aggregation at heterochromatin domains (e.g., mitophagy sites and telomeres), reinforcing chromatin compaction (Gan et al., 2015). KAP1 also functions as a scaffold, directing SETDB1 to specific genomic loci to catalyze H3K9me3 and silence retrotransposons (e.g., LINE-1) and imprinted genes. Thus, KAP1 SUMOylation is essential for chromatin compaction and repression of endogenous retroelements (Ivanov et al., 2007). However, KAP1’s repressive activity is attenuated upon DNA double-strand break (DSB) induction. ATM-mediated phosphorylation at Ser824 disrupts KAP1 SUMOylation and weakens its gene-silencing capacity (Lee et al., 2007). Protein phosphatase 1 (PP1), especially the α and β isoforms, reverses this effect by dephosphorylating Ser824 and promoting KAP1 SUMOylation, thereby restoring its repressive function (Li et al., 2010). In summary, KAP1 auto-SUMOylation promotes heterochromatin formation but is dynamically reversed during DNA damage repair (Li et al., 2010).

In addition to self-modification, KAP1 acts as a SUMO ligase for other substrates. For instance, it SUMOylates proliferating cell nuclear antigen (PCNA) via a PIP motif in its bromodomain, thereby preventing transcription-associated DNA breaks (Li et al., 2020). It also SUMOylates MORC2 at lysine 767 (K767), modulating its role in the DNA damage response; this modification is reversible by the deSUMOylase SENP1 (Zhang et al., 2023a).

Meanwhile, the N-terminal RING domain of KAP1 functions as an E3 ubiquitin ligase, influencing transcription factor stability and chromatin dynamics (Qin et al., 2021; Xue et al., 2024; Zhu and Xiao, 2024). For example, it promotes K48-linked polyubiquitination of p53, leading to its proteasomal degradation and attenuation of p53-mediated cell cycle arrest or apoptosis (Liu et al., 2022; Jang et al., 2024). KAP1 is also subject to self-ubiquitination, forming part of a negative feedback loop (Wang et al., 2020b). In addition, it can catalyze K63-linked polyubiquitination, which modulates the localization and stability of itself or associated proteins (Hua et al., 2024; Liu et al., 2025). Under stress, such auto-ubiquitination can drive liquid-liquid phase separation (LLPS), facilitating the formation of transcriptional repressor condensates (Ren et al., 2024). Moreover, KAP1 ubiquitinates DNA methyltransferase 3α (DNMT3A), affecting its enzymatic function and chromatin recruitment, and thereby modulating DNA methylation patterns (Li et al., 2024b).

SUMOylation at the C-terminus of KAP1 may trigger a conformational shift that occludes its N-terminal RING domain, thereby inhibiting ubiquitin ligase activity. This structural change may temporally uncouple transcriptional repression from protein degradation. Following DNA damage, SUMOylated KAP1 facilitates RNF4 recruitment, which catalyzes K63-linked ubiquitination of repair proteins, forming SUMO-ubiquitin hybrid chains that coordinate chromatin remodeling and DNA repair (Kuo et al., 2014). Under severe genotoxic stress, KAP1 promotes polyubiquitination and degradation of SIRT1, a class III histone deacetylase, thereby modulating its anti-apoptotic role in the DNA damage response (Ouyang et al., 2022).

In summary, KAP1 orchestrates chromatin silencing and DNA repair complex assembly via SUMOylation, while simultaneously regulating degradation of key factors and enzymatic activity through ubiquitination. These dual PTMs act as an integrated platform for chromatin dynamics and protein homeostasis, operating through site competition, signal amplification, and cooperative regulation.

### 3.4 Embryonic development and cell fate determination

Loss of maternal KAP1 results in early embryonic lethality, predominantly in male embryos (Cammas et al., 2000; Cheng, 2014; Sampath Kumar et al., 2017; Asimi et al., 2022), due to its essential role in maintaining epigenetic stability during the oocyte-to-embryo transition in mice (Messerschmidt et al., 2012). Studies employing conditional knockout models in both mice and cultured cells have demonstrated that KAP1 is critical for multiple developmental processes, including spermatogenesis, erythropoiesis, and the differentiation of T and B lymphocytes (**Table 1**). Beyond developmental regulation, KAP1 also contributes to immune homeostasis. It has been identified as a component of FOXP3–containing protein complexes and is known to enhance the suppressive function of regulatory T (Treg) cells. KAP1 is also implicated in immunoglobulin class-switch recombination, further linking it to immune regulation (Tanaka et al., 2018). In the central nervous system, Jakobsson et al. demonstrated that deletion of KAP1 in the mouse forebrain leads to pronounced behavioral changes, including increased anxiety-like behavior, heightened exploratory activity, and impaired spatial learning and memory in response to stress (Jakobsson et al., 2008). Notably, KAP1 deletion also disrupts olfactory neurogenesis (Pavlaki et al., 2018). Transcriptomic analyses revealed that several dysregulated genes in the hippocampus are imprinted genes, suggesting that KAP1 may play a role in the maintenance of genomic imprinting (Jakobsson et al., 2008). This hypothesis is supported by studies showing that KAP1 contributes to DNA methylation during early embryogenesis. It achieves this by recruiting DNA methyltransferases (DNMTs) to the imprinting control region (ICR), thereby reinforcing stable silencing of imprinted loci during key developmental windows (Haggerty et al., 2021).

**Table 1:** Mouse models illustrating the physiological functions of KAP1.

Animal model	Phenotype	Reference
KAP1 knockout mice	Embryonic lethal prior to gastrulation	(Cammas et al., 2000; Sampath Kumar et al., 2017)
Hemato-specific KAP1 knockout mice	Impaired erythropoiesis	(Barde et al., 2013; Hosoya et al., 2013; Cheng, 2014)
Chronic myeloid leukemia KAP1 knockout mice	Inhibits the progression of myeloid leukemia	(Morii et al., 2024)
T-cell-specific KAP1 knockout mice	Defective T-cell differentiation	(Chikuma et al., 2012; Sio et al., 2012)
B-cell-specific KAP1 knockout mice	Defective B-cell differentiation	(Santoni De Sio et al., 2012)
Tamoxifen-inducible-germ cell-lineage-specific KAP1 depletion mice	Impaired spermatogenesis	(Weber et al., 2002)
Liver-specific KAP1 knockout mice	Male-predominant steatosis and hepatic adenoma	(Bojkowska et al., 2012)
KAP1 knockout in mice forebrain	Anxiety-like-behavior and cognitive impairments	(Jakobsson et al., 2008)
KAP1 knockout mice in progeria models	Suppresses the induction of a senescence-associated secretory phenotype	(Santos and Gil, 2014)
Cell model	Phenotype	Reference
KAP1 knockdown in human aortic smooth muscle cells (HASMC)	Suppression of phenotypic transition dysregulation in vascular smooth muscle cells	(Liu et al., 2019)
KAP1 knockdown in porcine oocytes	Transcriptional activity of fertilized egg genes is blocked.	(Zhai et al., 2021)
KAP1 knockdown in sheep embryonic fibroblasts (SEF)	Disrupts gene expression of imprinted genes Igf2 and H19, which in turn affects epigenetic inheritance	(Luo et al., 2017)

## 4 KAP1 in viral infections: mechanisms and paradoxes

KAP1 is a well-established transcriptional repressor critical for silencing integrated retroviral genomes and maintaining herpesviral latency through heterochromatin formation (Iyengar and Farnham, 2011; Randolph et al., 2022). By recruiting epigenetic modifiers such as SETDB1, HP1, and histone deacetylases, KAP1 establishes transcriptionally repressive chromatin that inhibits viral gene expression (Cheng, 2014). Its role in controlling ERVs, HIV-1, Moloney Murine Leukemia Virus (MMLV), Prototype Foamy Virus (PFV), and herpesviruses such as EBV, KSHV, Human cytomegalovirus (HCMV), and Herpes simplex virus type 1 (HSV-1) is well documented and represents its primary antiviral function. Notably, reactivation from latency is often linked to post-translational modifications of KAP1, particularly Ser824 phosphorylation, which disrupts its repressive activity. In recent years, KAP1 has also been implicated in the regulation of other viruses, including SARS-CoV-2, Respiratory Syncytial Virus (RSV), IAV, HPV, Hepatitis C Virus (HCV), Merkel Cell Polyomavirus (MCPyV), and adenoviruses. Although these findings remain comparatively limited, they suggest broader roles for KAP1 in modulating viral replication and innate immune responses through transcriptional control mechanisms. This expanding scope does not diminish established function of KAP1 in retroviral and herpesviral silencing but rather highlights its emerging potential as a more universal regulator of host-virus interactions (Lork et al., 2021).

### 4.1 Epigenetic silencing of viral genomes

#### 4.1.1 KAP1-SETDB1-mediated silencing of ERVs and exogenous retroviruses

Retroviruses (RVs) belong to the *Retroviridae* family, a group of single-stranded, positive-sense, non-segmented RNA viruses that exclusively infect vertebrates (Krebs et al., 2021). RV replication involves reverse transcription of the viral RNA genome into double-stranded DNA, followed by integration of the resulting DNA into the host genome to form a provirus (Miyazato et al., 2016). While most RVs infect somatic cells, on rare occasions they infect germline cells (Greenwood et al., 2018). In such cases, the integrated provirus becomes a heritable component of the host genome and is transmitted vertically across generations, forming what are known as ERVs. ERVs, classified as long terminal repeat (LTR) retrotransposons, constitute approximately 9% of the human genome (Hughes, 2015) and are increasingly recognized for their roles in both human health and disease. The involvement of KAP1 in maintaining the transcriptional silencing and latency of both RVs and ERVs was identified early on (Fukuda and Shinkai, 2020; Randolph et al., 2022). Beyond ERVs, KAP1-mediated epigenetic silencing has also been observed in response to exogenous retroviruses, including HIV-1, MMLV, and PFV.

##### 4.1.1.1 ERVs

Under normal physiological conditions, ERVs are silenced through multiple epigenetic mechanisms, including heterochromatin formation, DNA methylation, and RNA transcript modifications (Chelmicki et al., 2021; Liu et al., 2021a). Among these, one of the most well-characterized silencing pathways involves KAP1-mediated heterochromatinization. In pluripotent embryonic stem cells (ESCs), KAP1 is recruited to ERVs via KRAB-ZFPs, where it cooperatively recruits SETDB1 and HP1 to establish a repressive chromatin environment. Similarly, in neural progenitor cells (NPCs), KAP1 mediates dynamic histone modifications to regulate ERVs transcriptional silencing (Rowe et al., 2010; Fasching et al., 2015; Brattås et al., 2017). KAP1 has also been shown to repress ERVs and zinc finger (ZNF) genes in differentiated human cell types such as HeLa, 293T, and peripheral blood mononuclear cells (PBMCs). This repression is closely linked to specific KAP1 binding sites and H3K9me3 enrichment, indicating that the KAP1-KRAB-ZFP (KZNF) complex contributes to genomic stability even in adult somatic cells (Tie et al., 2018). In addition to ERVs, KAP1 also regulates other human-specific endogenous retroelements (EREs) in ESCs. Its transcriptional repression function appears to be tightly coupled with KAP1-induced DNA methylation, although KAP1-mediated chromatin remodeling is essential for transcriptional regulation of EREs regardless of their DNA methylation status (Turelli et al., 2014).

Moreover, KAP1 cooperates with the human silencing hub (HUSH) complex to repress evolutionarily young LINE-1 elements and newly acquired genes rewired through retrotransposon-derived non-coding sequences (Robbez-Masson et al., 2018). Mechanistic studies have mapped KAP1-mediated transcriptional repression to a 190 bp sequence encoding the intracisternal A-particle (IAP) signal peptide in murine ESC and NPC models. Within this sequence, a 47 bp enhancer in the U3 region of the LTR has been identified as a key element for retrotransposon activity. Knockdown of KAP1 leads to derepression of IAPs, resulting in the loss of regulatory elements required for autonomous retrotransposition (Enriquez-Gasca et al., 2023).

Mounting evidence links aberrant expression of human endogenous retroviruses (HERVs) to autoimmune (Tovo et al., 2020b) and neurological disorders (Tovo et al., 2022). KAP1 and SETDB1, as central epigenetic repressors of HERV sequences, play important roles in immune regulation, neuronal differentiation, and synaptic function (Fasching et al., 2015; Kawabe and Stegmüller, 2021). For example, multiple sclerosis (MS) is a chronic inflammatory demyelinating disease of the central nervous system (CNS) (Dobson and Giovannoni, 2019). Interestingly, pregnancy has been shown to attenuate disease severity and reduce relapse frequency (Bove et al., 2024). Clinical studies have found significantly lower levels of HERV mRNA in the chorionic villi and basal plate tissues of pregnant women compared to non-pregnant individuals. However, concurrent impairment in KAP1 and SETDB1 expression suggests that dynamic regulation of these proteins may influence HERV activation and MS pathogenesis. Notably, the relationship between KAP1 expression and pregnancy-related hormonal changes remains to be elucidated (Tovo et al., 2023c).

In an experimental autoimmune encephalomyelitis (EAE) mouse model, knockdown of KAP1 led to increased dendritic cell (DC) counts in the spleen and enhanced T-cell-driven inflammatory responses, thereby exacerbating disease severity. Genome-wide analyses revealed that ERV elements in KAP1-deficient DCs suppressed the expression of adjacent inflammatory genes. Therefore, KAP1-mediated ERVs silencing is essential for maintaining proper immunoregulatory gene expression in DCs (Chikuma et al., 2021).

Chronic immune thrombocytopenia (CITP), an autoimmune disorder, has also been linked to dysregulated HERV activity (Volkmann et al., 2023). Studies in pediatric patients have shown that blood mRNA levels of KAP1 and SETDB1 are significantly elevated in CITP patients and positively correlated with expression of HERV-H and HERV-K, suggesting that KAP1 may contribute to the immunopathogenesis of CITP (Tovo et al., 2023a). Similarly, in children with autism spectrum disorders (ASD), elevated expression of HERV-H and HERV-K envelope genes was observed, along with increased KAP1 and SETDB1 mRNA levels compared to healthy controls. These findings imply that KAP1/SETDB1 may respond to environmental stimuli and reshape chromatin epigenetics, thereby participating in the etiology of ASD (Tovo et al., 2022).

##### 4.1.1.2 HIV-1

HIV-1 is the causative agent of acquired immunodeficiency syndrome (AIDS). It attacks the immune system by depleting CD4^+^T lymphocytes, leading to immunodeficiency and increased susceptibility to opportunistic infections and malignancies (Swinkels et al., 2025). A major obstacle to HIV-1 eradication is the persistence of transcriptionally silent proviruses, referred to as latent HIV-1, within long-lived host reservoirs. To address this challenge, the “shock-and-kill” strategy has been widely explored, which involves reactivating latent proviruses (“shock”) followed by immune- or drug-mediated clearance of the reactivated cells (“kill”) (Chou et al., 2024). Therefore, the development of effective latency-reversing agents (LRAs) remains a key objective in HIV-1 therapy.

The role of KAP1 in HIV-1 infection remains controversial, particularly with regard to whether it promotes viral transcription or enforces latency. Early studies suggested that KAP1 restricts HIV-1 by interacting with viral integrase and preventing proviral integration into host chromatin (Allouch et al., 2011). KAP1 has also been implicated in the regulation of HIV-1 transcription (McNamara et al., 2016b), where it recruits inactive P-TEFb to the HIV-1 LTR to enforce transcriptional pausing under basal conditions but at the same time primes the promoter for rapid activation upon stimulation. In CD4^+^T cells, KAP1 has been associated with both transcriptional activation and repression, contributing to the ongoing debate (Morton et al., 2019; Taura et al., 2019).

Current evidence suggests that KAP1 plays a central role in promoting HIV-1 latency through transcriptional repression mechanisms. Ma and colleagues reported that KAP1 mediates SUMOylation of the CDK9 subunit of P-TEFb at lysine residues K44, K56, and K68. This modification reduces CDK9 kinase activity and impairs P-TEFb assembly by disrupting its interaction with Cyclin T, thereby repressing transcriptional elongation and promoting HIV-1 latency (Ma et al., 2019). These findings highlight the dual role of KAP1 as both a SUMO ligase and a chromatin-based transcriptional corepressor (Ait-Ammar et al., 2021).

KAP1 has also been shown to interact with the viral transactivator Tat, facilitating its degradation, and with CTIP2, a key epigenetic silencing factor. In microglial cells, Tat and CTIP2 compete for binding to KAP1, suggesting that KAP1 contributes to the establishment and maintenance of latency through cell type-specific molecular interactions (Ait-Ammar et al., 2021).

A genome-wide CRISPR knockdown screen identified ZNF304, a KRAB-domain-containing zinc finger protein, as a host factor that promotes HIV-1 latency. ZNF304 recruits KAP1 to the viral promoter, where they together facilitate the assembly of heterochromatin-associated histone methyltransferases (KMTs) and polycomb repressive complexes (PRCs), enforcing transcriptional silencing. Loss of ZNF304 leads to a marked reduction in repressive histone modifications at the HIV-1 promoter–including H3K9me3, H3K27me3, and H2AK119ub–as well as diminished KAP1 recruitment, resulting in increased HIV-1 gene expression. These findings underscore the cooperative role of ZNF304 and KAP1 in sustaining viral latency (Krasnopolsky et al., 2020).

Contrastingly, recent studies have revealed a potential transcriptional activator role for KAP1 under certain conditions. Acute depletion of KAP1 using a chemical genetics approach partially reduced HIV-1 promoter activity in response to activation stimuli. This phenotype was rescued by reintroducing exogenous KAP1, implicating KAP1 as a targeted transcriptional co-activator. Structural mapping further identified the RING finger domain and an intrinsically disordered region of KAP1 as essential for this activating function (Morton et al., 2019; Randolph et al., 2024). Moreover, exposure to cocaine has been shown to promote phosphorylation of KAP1 at Ser824 via DNA-dependent protein kinase (DNA-PK), converting KAP1 from a repressor to an activator of HIV-1 transcription. This observation may help explain the link between substance abuse and poor viral control in HIV-infected individuals (Sharma et al., 2024).

In summary, the role of KAP1 in HIV-1 infection is multifaceted and context-dependent. While it is clearly involved in establishing and maintaining latency through SUMOylation and transcriptional repression, under certain conditions KAP1 can also facilitate viral gene activation. The precise mechanisms and regulatory switches governing these opposing roles remain incompletely understood and warrant further investigation.

##### 4.1.1.3 MMLV

MMLV is a retrovirus known for its ability to infect mice and other vertebrates, where it can contribute to oncogenesis. Although MMLV cannot replicate in embryonic carcinoma (EC) cells or embryonic stem cells (ESCs) (Linney et al., 1984), it can successfully integrate into the host genome as proviral DNA, which is subsequently transcriptionally silenced (Wang et al., 2014).

The key regulatory element responsible for this silencing is the proline primer binding site (PBS) of MMLV (Yamauchi et al., 1995), which is complementary to host proline tRNA. This complementarity facilitates the initiation of negative-strand DNA synthesis and is thought to contribute to transcriptional repression. Further studies revealed that KAP1 is recruited to the repressor binding site (RBS) of MMLV, where it coordinates with HP1, SETDB1, and the NuRD complex to establish transcriptional silencing of the integrated provirus (Wolf et al., 2008). A central player in this process is ZFP809, a zinc finger protein that facilitates the recruitment of KAP1 and its associated silencing machinery to the MMLV proviral DNA. The interaction between KAP1 and ZFP809 is critical for chromatin and DNA modifications that enforce the silent state, and KAP1 also helps stabilize ZFP809 protein levels, further supporting sustained repression (Wolf and Goff, 2007). Notably, this mechanism appears to be specific to ESCs, and the role of KAP1 in regulating ZFP809 may differ in differentiated cells (Wang and Goff, 2017). Subsequent studies identified another DNA-binding factor-YY1-as a sequence-specific mediator that links KAP1 to the MMLV provirus. KAP1 interacts with the acidic domain 1 and the zinc finger domain of YY1 through its RBCC region (Lee et al., 2018). In ESCs, SUMOylation of KAP1 at lysine residue K779 was found to be essential for its gene-silencing activity against MMLV, reinforcing the importance of post-translational modification in KAP1 function (Lee et al., 2018).

Recent findings have further expanded the understanding of MMLV repression by implicating the chromatin remodeler Smarcad1. This protein interacts with KAP1 and facilitates the deposition of the histone variant H3.3 at proviral integration sites. H3.3 incorporation is thought to modulate chromatin dynamics, contributing to stable repression of MMLV transcription (Elsässer et al., 2015). Depletion of either Smarcad1 or KAP1 leads to derepression of MMLV, indicating that the two proteins function cooperatively (Bren et al., 2024). However, the precise mechanisms by which KAP1 and Smarcad1 coordinate the regulation of proviral gene expression and genome integrity remain to be fully elucidated.

##### 4.1.1.4 PFV

PFV is a complex retrovirus that, despite its strong cytopathic effects in cultured cells, does not cause overt pathology in its natural host. Instead, PFV establishes a lifelong latent infection (Wei et al., 2022). Similar to other retroviruses, PFV latency is regulated epigenetically. KAP1 contributes to this process by maintaining trimethylation of histone H3 lysine 9 (H3K9me3) at the long terminal repeat (LTR) promoter of the viral genome. This epigenetic mark facilitates the recruitment of HP1, forming a repressive chromatin environment that supports the maintenance of PFV latency (Yuan et al., 2021).

#### 4.1.2 Herpesvirus latency maintenance and KAP1 phosphorylation switch

Herpesviruses are nearly ubiquitous in the human population and are classified into three major subfamilies-α, β, and γ-based on their biological properties and host cell tropism. A hallmark of all herpesviruses is their ability to establish latency within specific cell types (Kanda, 2018), remaining transcriptionally silent or quiescent for extended periods (Bhaduri-McIntosh and Rousseau, 2024). Periodic reactivation into the lytic (productive) cycle enables viral replication and transmission. While α- and β-herpesviruses primarily cause disease during the lytic phase, γ-herpesviruses are associated with oncogenic potential, particularly in latently infected cells (Cohen, 2020). Transcriptional silencing during latency is essential for long-term viral persistence. Analogous to its role in retroviral repression, KAP1 contributes to the maintenance of herpesvirus latency by promoting constitutive heterochromatin formation on the viral genome. Through its interaction with epigenetic modifiers, KAP1 enforces silencing of lytic genes, thus stabilizing the latent state (Li et al., 2018, 2019; Burton et al., 2021). SUMOylation of KAP1 is a prerequisite for its gene-silencing function. However, upon entry into the lytic cycle, this repressive modification is dynamically replaced by phosphorylation, typically at serine residue 824 (Ser824). Although the specific kinases involved vary among different herpesviruses, both host and viral kinases are capable of inducing this phosphorylation. The phosphorylation of KAP1 at Ser824 acts as a molecular “switch” that disrupts its repressor function, thereby enabling reactivation of viral gene expression and progression to productive infection.

This phosphorylation-dependent regulatory mechanism positions KAP1 as a potential therapeutic target, and pharmacological induction of KAP1 Ser824 phosphorylation could potentially be explored to reactivate latent herpesviruses (Bhaduri-McIntosh and Rousseau, 2024).

##### 4.1.2.1 EBV

EBV, a member of the *Lymphocryptovirus* genus within the *Herpesviridae* family, is the etiological agent of infectious mononucleosis (IM) and has been strongly implicated in the development of nasopharyngeal carcinoma, various pediatric lymphomas, and autoimmune diseases (Damania et al., 2022). EBV infects more than 95% of adults worldwide and establishes lifelong latency in host cells (Soldan and Lieberman, 2023).

The maintenance of EBV latency involves multiple layers of transcriptional repression in which KAP1 plays a central role. KRAB-ZFPs suppress expression of EBV lytic genes by recruiting KAP1, thereby contributing to the silencing of the viral genome (Li et al., 2018). In addition, the EBV latency protein Epstein-Barr nuclear antigen 1 (EBNA1) can recruit KAP1 via its SUMO-interacting motif 3 (SIM3), further supporting viral latency (Wang et al., 2020a). EBV replication is governed by a tripartite helicase-primase complex composed of the deconjugating enzyme BBLF4, the primase BSLF1, and replication factors BBLF2/3 (Thierry et al., 2015). Yeast two-hybrid screening and co-immunoprecipitation assays using BBLF2/3 as bait identified an interaction between the DNA-binding zinc finger protein ZBRK1, its co-repressor KAP1, and BBLF2/3. ZBRK1 was shown to bind to the oriLyt enhancer–EBV’s lytic origin of replication-indicating that the ZBRK1-KAP1 complex serves as a key regulator of EBV replication control at this site (Xu et al., 2023).

KAP1 also binds to the *oriLyt* and immediate early gene promoters in a CTAR3-dependent manner. CTAR3 is a signaling domain within EBV’s latent membrane protein, and it facilitates SUMOylation of KAP1, reinforcing its transcriptional repressor function and contributing to maintenance of EBV latency (Bentz et al., 2015). Furthermore, the interferon-inducible protein IFI16 is required for latency and directly interacts with KAP1 to reinforce repression of viral gene expression (Pisano et al., 2017; Xu et al., 2022a). A critical regulatory switch in EBV latency is the promoter of BZLF1 (BamHI Z left fragment 1), a master transcriptional activator often referred to as the “lytic switch”. Its activation initiates the transition from latency to productive replication (Li et al., 2019). SUMO proteomic analyses revealed that the TRIM24/KAP1/TRIM33 complex suppresses BZLF1 expression during latent infection, suggesting that this multi-protein repressor complex may serve as a cellular defense mechanism against EBV lytic reactivation (De La Cruz-Herrera et al., 2023).

To overcome latency, EBV encodes several factors that modulate host signaling (Leonardi et al., 2022). The viral transactivator BZLF1 is essential for reactivation and is implicated in EBV-associated tumorigenesis (Germini et al., 2020). BZLF1 expression is maintained by the viral protein kinase (vPK), which activates host phosphoinositide 3-kinase (PI3K) signaling and induces phosphorylation of KAP1 at Ser824 (Xu et al., 2023). This modification disables KAP1’s repressive activity, facilitating BZLF1-driven amplification of the lytic cascade and promoting viral replication. In this context, KAP1’s latency-maintaining function is hijacked by viral signaling to enable reactivation (Li et al., 2019; Xu et al., 2022a).

Other stressors can similarly subvert KAP1 function. Chloroquine, a known agonist of ATM kinase and antimalarial drug, has been shown to induce EBV reactivation in patients coinfected with *Plasmodium falciparum*, likely via ATM-mediated phosphorylation of KAP1 at Ser824 (Li et al., 2017). In addition, EBV activates thioredoxin-interacting protein (TXNIP), a regulator of the NLRP3 inflammasome. TXNIP-induced activation of caspase-1 leads to degradation of KAP1 in certain cell populations, disrupting transcriptional silencing and triggering EBV reactivation (Burton et al., 2020). Together, these findings demonstrate that KAP1 is a key regulator of EBV latency and that its functional inactivation through phosphorylation or degradation represents a convergent mechanism exploited by EBV to initiate lytic replication.

##### 4.1.2.2 KSHV

KSHV is implicated in the pathogenesis of several malignancies, including Kaposi’s sarcoma (KS), primary effusion lymphoma (PEL), and multicentric Castleman’s disease. Like other herpesviruses, KSHV alternates between latent and lytic replication phases (Broussard and Damania, 2020). Understanding the molecular mechanisms that regulate the transition from latency to lytic reactivation is critical for controlling viral dissemination and developing targeted antiviral strategies (Chang et al., 2009; Yan et al., 2024).

A key latency-associated protein encoded by KSHV is the latency-associated nuclear antigen (LANA), which possesses a SUMO-2–interacting motif that enables it to engage KAP1 through SUMOylation. This interaction promotes chromatin remodeling and contributes to the silencing of lytic genes during early stages of primary infection (Sun et al., 2014). Additionally, nuclear factor erythroid 2-related factor 2 (NRF2) has been shown to facilitate the interaction between LANA and KAP1, reinforcing transcriptional repression of lytic genes (Gjyshi et al., 2015).

The cellular transcription factor STAT3 also plays a role in maintaining KSHV latency. Suppression of STAT3 leads to downregulation of KAP1 and consequently activates the viral replication and transcription activator (RTA), highlighting a STAT3-KAP1 axis that regulates the responsiveness of latently infected cells to lytic stimuli (King et al., 2015). Cai and colleagues reported that KSHV induces the accumulation of hypoxia-inducible factor-1α (HIF-1α) during latency. Under hypoxic stress, HIF-1α facilitates reactivation of the KSHV lytic cycle by disrupting the repressive complex formed between KAP1 and Sin3A at the SIM motif of LANA. This dissociation reduces KAP1 SUMOylation, enabling transcriptional activation of lytic genes. Furthermore, the RTA promoter contains overlapping binding sites for RBP-Jκ and HIF-1α (known as RBS and HRE, respectively). Inhibition of KAP1 enhances the binding of RBP-Jκ-HIF-1α complexes at this promoter region, suggesting that KAP1 occupancy at the RTA promoter is essential for suppressing lytic reactivation under normoxic conditions (Zhang et al., 2014).

As with other herpesviruses, phosphorylation of KAP1 at Ser824 by viral protein kinases induces chromatin remodeling and activates lytic gene transcription, thereby promoting the switch from latency to replication (Ali et al., 2022). The viral protein kaposin B contributes to the chronic inflammatory environment characteristic of KS by activating the host kinase MK2 (MAPKAPK2), which in turn phosphorylates KAP1 and facilitates lytic reactivation (King, 2013). In addition to modulating KAP1 phosphorylation, KSHV has evolved strategies to counteract KAP1-mediated proteasomal degradation of key host transcriptional regulators. For instance, TFIIB, a core component of the RNAPII transcriptional machinery, is typically cleaved by caspase-3 and further degraded via KAP1-mediated ubiquitination in response to cellular stress. However, KSHV impairs this degradation process, thereby preserving TFIIB and supporting viral gene expression (King, 2013).

Together, these findings demonstrate that KSHV not only hijacks the phosphorylation switch of KAP1 to escape latency but also manipulates multiple host signaling pathways to overcome KAP1-mediated transcriptional repression, enabling efficient viral replication and immune evasion.

##### 4.1.2.3 HCMV

HCMV infection is a widespread β-herpesvirus that establishes lifelong latency, primarily within hematopoietic stem cells (HSCs) (Griffiths and Reeves, 2021). In immunocompetent individuals, viral latency is typically well controlled; however, reactivation in immunocompromised patients, such as organ transplant recipients, can result in life-threatening disease (Gugliesi et al., 2021).

In human myeloid leukemia cells (Kasumi-3), HCMV gene expression initially becomes transiently activated before being repressed, reflecting a myeloid lineage-specific host defense mechanism that enforces viral transcriptional silencing (Rozman et al., 2022). Tumor necrosis factor alpha (TNF-α) has been shown to trigger HCMV reactivation, and this process is tightly linked to the gene-silencing function of KAP1 (Forte et al., 2018). The RNA-binding protein SERPINE1 mRNA-binding protein 1 (SERBP1) functions as a scaffold that facilitates the recruitment of KAP1 to the viral genome, promoting transcriptional repression of the major immediate-early promoter (MIEP) (Reichel et al., 2018). Genetic depletion of KAP1 in CD34^+^ cord blood-derived progenitor cells leads to enhanced expression of early and late HCMV genes, confirming that KAP1 is essential for the maintenance of viral latency (Poole and Sinclair, 2022). Upon differentiation of dendritic cells (DCs), which triggers HCMV reactivation, KAP1 remains associated with the viral genome; however, SETDB1 and H3K9me3 occupancy at viral promoters is diminished, thereby allowing escape from the latent state (Poole and Sinclair, 2022). Additionally, phosphorylation of KAP1 at Ser824-induced by mTOR signaling or exposure to chloroquine-has been shown to disrupt its repressive function, further supporting the notion that Ser824 phosphorylation serves as a molecular switch enabling HCMV reactivation (Rauwel et al., 2015).

##### 4.1.2.4 HSV-1

Among α-herpesviruses, HSV-1 is one of the most prevalent human pathogens, establishing lifelong latency primarily in sensory neurons (Harrison and Jones, 2022). Reactivation from latency leads to recurrent mucocutaneous lesions and, in severe cases, life-threatening encephalitis (Ak et al., 2025). During the early stages of HSV-1 infection, the host mounts an epigenetic defense response characterized by the deposition of repressive histone marks, particularly H3K9me3, on the viral genome, leading to suppression of lytic gene transcription (Tsai et al., 2022). As viral reactivation proceeds, the integrin-linked kinase (ILK)-activated downstream of phosphoinositide 3-kinase (PI3K)-stimulates protein kinase B (PKB/Akt) and facilitates productive viral infection. ILK opposes the activity of histone methyltransferase SUV39H1 and KAP1, both of which contribute to silencing of HSV-1 lytic genes. Knockdown of ILK results in reduced phosphorylation of KAP1 at Ser473 and Ser824, indicating that ILK enhances KAP1 phosphorylation to overcome transcriptional repression of lytic genes (Tsai et al., 2022). These findings suggest that the ILK-KAP1 axis represents a critical regulatory node for HSV-1 reactivation and may serve as a potential therapeutic target.

#### 4.1.3 Transcriptional repression by KAP1 in emerging viral infections

Beyond its well-characterized roles in retroviruses and herpesviruses, KAP1 has recently garnered attention for its potential involvement in other viral infections. In several emerging systems, KAP1 is proposed to act as a transcriptional repressor through epigenetic mechanisms, suggesting a broader regulatory scope that remains to be fully elucidated.

##### 4.1.3.1 HPV

HPV is small double-stranded DNA virus of the Papillomaviridae family that infects epithelial cells. While most HPV infections are transient and asymptomatic, persistent infection with high-risk types such as HPV-16 and HPV-18 is strongly associated with the development of cervical cancer and other anogenital and oropharyngeal malignancies (Liu et al., 2015; Schichl and Doorbar, 2025). KAP1 functions as a co-repressor of E2F, a key transcription factor involved in HPV gene regulation. By promoting chromatin remodeling and transcriptional silencing, KAP1 influences the expression of viral oncogenes E6 and E7, which are critical for HPV-mediated oncogenesis. These oncogenes inactivate tumor suppressor proteins p53 and retinoblastoma (Rb), leading to dysregulated cell cycle progression and enhanced cellular proliferation. Through its epigenetic regulatory activity, KAP1 may thus contribute to viral persistence and the progression of HPV-associated malignancies (Gao et al., 2020).

##### 4.1.3.2 HCV

HCV is an enveloped, positive-stranded RNA virus of the Flaviviridae family that infects hepatocytes. Globally, chronic HCV infection affects more than 50 million people and is a leading cause of liver fibrosis, cirrhosis, and hepatocellular carcinoma (Spearman et al., 2019). Several TRIM family proteins have been shown to restrict HCV by regulating viral transcription, replication, and assembly. As a key member of the TRIM family, KAP1 plays a regulatory role during HCV infection by modulating host genomic elements (Cao et al., 2024). Upon HCV infection, KAP1 appears to reduce its transcriptional repression activity toward human HERVs, particularly HERV-H-pol and HERV-K-pol, leading to their aberrant upregulation. This derepression may influence viral replication dynamics and promote the aggregation of viral particles (Weber et al., 2021). These findings suggest that KAP1 acts as a host epigenetic regulator whose functional alteration during HCV infection may contribute to viral persistence, making it a potential therapeutic target for HCV intervention (Tovo et al., 2020a).

##### 4.1.3.3 MCPyV

MCPyV is a small double-stranded DNA virus of the Polyomaviridae family and the only polyomavirus conclusively linked to human cancer. MCPyV is clonally integrated in the majority of Merkel cell carcinoma cases, an aggressive neuroendocrine skin cancer with high morbidity and mortality (Harms et al., 2018). KAP1 plays a critical regulatory role in MCPyV infection. Upon expression of MCPyV large and small tumor antigens (LT-Ag and ST-Ag) in normal human dermal fibroblasts (NHDFs), KAP1 undergoes phosphorylation at Ser824–a modification associated with the loss of its transcriptional repression activity. This phosphorylation event correlates with a marked increase in viral replication, indicating that KAP1 normally acts as a restriction factor during MCPyV infection. Its functional inactivation through phosphorylation enables viral gene expression and propagation, highlighting KAP1 as a key host factor modulated by MCPyV to facilitate productive infection (Siebels et al., 2020).

##### 4.1.3.4 Human adenoviruses

HAdVs are non-enveloped, double-stranded DNA viruses of the Adenoviridae family that cause a broad spectrum of diseases, ranging from respiratory and ocular infections to gastroenteritis. While typically self-limiting in immunocompetent hosts, HAdV infections can lead to severe or disseminated disease in immunocompromised individuals (Lion, 2014; Kajon, 2024). In human adenovirus (HAdV) infections, KAP1 undergoes de-SUMOylation to relieve epigenetic repression and promote viral gene expression. This process is also linked to enhanced SUMOylation of the viral protein E1B-55K, facilitating viral replication (Bürck et al., 2016).

### 4.2 Effects of KAP1 ubiquitination/SUMOylation on viral proteins and antiviral innate immune regulation

#### 4.2.1 Regulation of viral and host proteins via PTMs

KAP1 not only helps maintain heterochromatin at the PFV promoter by regulating H3K9me3, but also directly targets viral proteins for degradation. Its RBCC domain binds to the PFV transactivator Tas and promotes its ubiquitin-mediated degradation, thereby contributing to the establishment of viral latency. This dual mechanism highlights KAP1’s role as both an epigenetic repressor and a post-translational regulator during PFV infection (Yuan et al., 2021).

In hepatitis B virus (HBV), the multifunctional regulatory protein HBx modulates viral replication and host responses. KAP1, recruited by glycerol-3-phosphate dehydrogenase 2 (GPD2), facilitates HBx degradation via ubiquitination, thereby limiting HBV replication and potentially slowing liver disease progression (Liu et al., 2023a).

SARS-CoV-2, the causative agent of COVID-19, is a positive-sense, single-stranded RNA virus of the *Betacoronavirus* genus. Since its emergence in late 2019, it has caused a global pandemic with significant morbidity and mortality (Garigliany et al., 2020; V’kovski et al., 2021; Steiner et al., 2024). During SARS-CoV-2 infection, KAP1 exerts antiviral effects at both the transcriptional and protein levels. Specifically, KAP1 catalyzes SUMOylation of the viral nucleocapsid protein (SARS2-NP) at lysine 65, promoting its homo-oligomerization, RNA binding, and liquid-liquid phase separation (LLPS)-processes that impair the host innate immune response. Blocking this SUMOylation or LLPS formation may restore antiviral immunity and inhibit viral replication, offering a potential therapeutic strategy against COVID-19 (Zhang et al., 2021).

Porcine reproductive and respiratory syndrome virus (PRRSV) is a positive-stranded RNA virus of the Arteriviridae family and one of the most economically devastating pathogens in the swine industry. PRRSV infection causes reproductive failure in sows and respiratory disease in piglets, resulting in major global losses in pig production (Gao and Wen, 2025). KAP1 also plays a proviral role in PRRSV infection by enhancing viral replication through regulation of K63-linked ubiquitination of the envelope glycoprotein GP4. Knockdown of KAP1 significantly suppresses PRRSV replication, identifying it as a critical host factor in the viral life cycle (Cui et al., 2023).

Interestingly, KAP1 exhibits cell-type-specific regulatory effects on the zinc finger protein ZFP809. In embryonic stem cells (ESCs), KAP1 stabilizes ZFP809 and facilitates transcriptional repression of retroelements. However, in differentiated cells, KAP1 promotes ubiquitin-mediated degradation of ZFP809 via a C-terminal sequence containing lysine 391 (Wang and Goff, 2017). This functional switch-from transcriptional silencing in ESCs to ubiquitination-mediated degradation in somatic cells-may explain why MMLV replication is restricted in ESCs but not in differentiated cells (Wang and Goff, 2017).

#### 4.2.2 Effects on host antiviral innate immune signaling

When host cells encounter viral pathogens, pattern recognition receptors (PRRs) such as retinoic acid-inducible gene I (RIG-I) detect viral RNA and initiate signaling cascades (Xu et al., 2022b) involving MAVS, TBK1, and downstream transcription factors IRF3 and IRF7. These pathways culminate in the induction of type I interferons (IFNs), which are essential for establishing an antiviral state (Li et al., 2024a). Post-translational modifications, particularly ubiquitination and SUMOylation, play a crucial role in modulating these pathways, often through cross-regulatory mechanisms. While these modifications enhance innate signaling, some viruses exploit the same systems to suppress immune responses (Xiao et al., 2024; Yoneyama et al., 2024).

In this context, KAP1 directly interacts with TBK1, catalyzing K63-linked ubiquitination to amplify TBK1 activity and promote downstream type I IFN production. These findings identify TBK1 as a direct substrate of KAP1 and suggest that KAP1 can act as a positive regulator of antiviral responses through ubiquitin signaling (Hua et al., 2024).

Conversely, KAP1 has been shown to negatively regulate MAVS-mediated immune activation. Overexpression of KAP1 suppresses, while its knockdown enhances, MAVS-induced production of type I IFNs and proinflammatory cytokines (Liu et al., 2023b). Mechanistically, KAP1 mediates K48-linked polyubiquitination of MAVS, targeting it for proteasomal degradation. This process depends on cysteine residues C65 and C68 within the RING domain of KAP1 and lysines K7, K10, K371, K420, and K500 of MAVS (Chen et al., 2023).

Further downstream, KAP1 also modulates IRF7, a master regulator of type I IFN responses. Through its RING domain, KAP1 catalyzes the SUMOylation of IRF7, reducing its transcriptional activity and suppressing IFNB1 expression. The RNA-binding protein RBM45 has been implicated in facilitating the recruitment of KAP1 to IRF7, suggesting a broader regulatory network in play. KAP1 thus serves as a negative regulator of IRF7-dependent transcriptional responses (Liang et al., 2011).

Upstream of these events, KAP1 is a key modulator within this landscape. UBR5, an E3 ubiquitin ligase, has been identified as a positive regulator of RLR signaling. It promotes K63-linked ubiquitination of KAP1, which inhibits its intramolecular SUMOylation and relieves KAP1-mediated transcriptional repression on RLR genes. Thus, UBR5-mediated ubiquitination of KAP1 enhances RIG-I signaling by disinhibiting antiviral gene expression (Yang et al., 2024).

In addition to the RLR pathway, KAP1 has been shown to suppress NF-κB signaling by SUMOylating TRAF6, which interferes with its auto-ubiquitination and activation during innate immune responses (Yang et al., 2025). This indicates that KAP1 not only regulates antiviral signaling but also plays a role in modulating inflammation.

Finally, a complex and reciprocal interaction between KAP1 and the interferon-stimulated gene XAF1 further illustrates the regulatory versatility of KAP1. Upon RNA virus infection, MAVS recruits TBK1 and XAF1. TBK1 phosphorylates XAF1 at Ser252, enabling its nuclear translocation, where it partners with IRF1 to guide KAP1 to antiviral gene loci (Kuang et al., 2023). XAF1 targets the PHD domain of KAP1 for de-SUMOylation, enhancing chromatin accessibility and driving robust expression of antiviral genes (Kuang et al., 2023). Interestingly, KAP1, in turn, promotes the K48-linked polyubiquitination and degradation of XAF1 in an apparent feedback mechanism to restore its own repressive function-though the precise relevance of this counter-regulation in antiviral contexts remains to be fully elucidated (Jang et al., 2024) (**Table 2**).

**Table 2:** Roles of KAP1 in regulating host antiviral innate immunity.

Target protein	KAP1-associated function	Regulatory outcome
TBK1	KAP1 promotes K63-linked ubiquitination of TBK1, enhancing its protein expression and antiviral activity.	Promotes host innate immune signaling
MAVS	KAP1 induces K48-linked ubiquitination of MAVS, leading to its proteasomal degradation and weakened signaling.	Suppresses host antiviral signaling
IRF7	KAP1 promotes SUMOylation and degradation of IRF7, reducing expression of antiviral genes.	Suppresses host antiviral gene expression
UBR5	UBR5 modifies KAP1 via K63-linked ubiquitination, preventing its SUMOylation and relieving transcriptional repression on RLRs.	Promotes host innate immune activation
TRAF6	KAP1 mediates SUMOylation of TRAF6 and inhibits its ubiquitination-mediated NF-κB activation.	Suppresses host inflammatory responses
XAF1	XAF1 induces de-SUMOylation of KAP1, promoting expression of antiviral genes.	Promotes host antiviral gene expression

#### 4.2.3 KAP1 in virus-induced immune regulation

Although mechanistic insights remain limited, accumulating evidence suggests that KAP1 may participate in shaping host immune responses during infection with various RNA viruses. These roles include modulating interferon signaling, inflammatory responses, and cellular processes such as mitophagy, thereby contributing to immune defense or facilitating immune evasion depending on the viral context.

##### 4.2.3.1 SARS-CoV-2

A 2022 clinical study involving 188 mildly and 142 severely infected patients reported a significant reduction in KAP1 mRNA expression in both groups, with a more marked decrease in severe cases, suggesting that KAP1 downregulation may be associated with disease severity (Lu et al., 2020; Tavakoli et al., 2022; Acharya et al., 2023). Additional pediatric data showed higher expression of interferon-stimulated genes (ISGs) in children with mild symptoms, whereas those with severe disease or multisystem inflammatory syndrome in children (MIS-C) exhibited reduced interferon levels. The expression trends of KAP1, SETDB1, and HERV-related transcripts followed similar patterns, implicating KAP1 in SARS-CoV-2-associated innate immunity and transcriptional regulation (Tovo et al., 2021; Pellegrina et al., 2022). Furthermore, a genome-wide CRISPR-Cas9 screen identified KAP1, along with TRIM33 and EHMT1/2, as proviral host factors that may facilitate SARS-CoV-2 transcription and viral particle formation (Sakai et al., 2024).

##### 4.2.3.2 RSV

RSV is an enveloped, negative-stranded RNA virus of the Pneumoviridae family and a leading cause of acute lower respiratory tract infections in infants and young children. Globally, RSV is responsible for substantial pediatric morbidity and mortality, particularly in severe bronchiolitis cases (Wang et al., 2024). In pediatric patients with severe RSV-induced bronchiolitis, expression levels of KAP1 and SETDB1 were positively correlated with type I interferon production, suggesting a protective role for this complex in antiviral immune responses. While direct mechanistic evidence remains limited, these correlations imply that KAP1 may contribute to innate immune activation during RSV infection (Liang et al., 2011; Tovo et al., 2023b).

##### 4.2.3.3 IAV

IAV is an enveloped, negative-stranded RNA virus of the Orthomyxoviridae family that causes seasonal epidemics and occasional pandemics in humans. Beyond acute respiratory illness, severe IAV infection can trigger excessive inflammation and cytokine storm, contributing to high morbidity and mortality worldwide (Wildenbeest et al., 2024; Zhao et al., 2024). In IAV-infected cells, KAP1 undergoes de-SUMOylation, disrupting its interaction with SETDB1 and reducing H3K9me3 deposition at transposable element (TE) loci (Chang et al., 2021; Feng et al., 2022). This derepression of ERVs elements leads to the release of ERV-derived RNAs, which are sensed as host-encoded pathogen-associated molecular patterns (PAMPs), thereby enhancing interferon-mediated antiviral signaling through the RIG-I/MAVS/TBK1/JAK1 axis. The Schmidt group proposed that KAP1 acts as a regulatory switch: its SUMOylation status governs ERV repression and subsequent immune activation (Schmidt et al., 2019). Additionally, Krischuns et al. reported that infection with highly pathogenic avian influenza virus (HPAIV), including subtypes H1N1, H7N7, H7N9, and H5N1, induced phosphorylation of KAP1 at Ser473. This modification altered KAP1 interaction with HP1 and activated the PKR/MAPK/MSK1 signaling cascade, contributing to elevated levels of IFN-β, IL-6, and IL-8 and amplifying the inflammatory response (Krischuns et al., 2018).

##### 4.2.3.4 Porcine epidemic diarrhea virus

PEDV is an enveloped, positive-stranded RNA virus of the Coronaviridae family, Alphacoronavirus genus, that causes acute enteric disease in swine. PEDV infection leads to severe diarrhea, vomiting, and dehydration, with particularly high mortality in neonatal piglets, making it a major pathogen of economic concern in the swine industry (Crawford et al., 2016; Jung et al., 2020). In PEDV infection, KAP1 appears to be hijacked by the virus to facilitate immune evasion. PEDV, a member of the *Alphacoronavirus* genus, suppresses host antiviral responses by inhibiting IRF7 phosphorylation[]. In addition, the PEDV nucleocapsid (PEDV-N) protein promotes the upregulation of host KAP1, which in turn induces mitophagy and suppresses the JAK-STAT1 signaling pathway, ultimately enhancing viral replication. Thus, PEDV exploits KAP1-mediated pathways to attenuate host immunity and promote viral proliferation (Li et al., 2024c).

### 4.3 Additional regulatory roles of KAP1 in viral infection

Beyond its transcriptional silencing activity, KAP1 also regulates viral replication through mechanisms involving virus-host interaction dynamics (Pellegrina et al., 2022). For example, SARS-CoV-2 enters host cells via the angiotensin-converting enzyme 2 (ACE2) receptor (Jackson et al., 2022), whose expression level directly influences host susceptibility and disease severity. KAP1 is co-expressed with ACE2 in type II alveolar epithelial cells (Ashraf et al., 2021; Jackson et al., 2022). Knockdown of KAP1 leads to ACE2 upregulation, thereby enhancing SARS-CoV-2 entry into both A549 lung carcinoma cells and primary human alveolar epithelial cells (HPAEpiC)—an effect partially reversed by granzyme B inhibition (Wang et al., 2021). Additionally, KAP1 depletion increases IFN-γ receptor 2 (IFNGR2) expression and promotes interferon-gamma (IFN-γ) secretion, further elevating ACE2 levels. These findings suggest that KAP1 may restrict viral entry by suppressing ACE2 expression, thereby contributing to host antiviral defense (Wang et al., 2021). Similarly, ASFV manipulates host coagulation pathways via KAP1. ASFV infection is associated with disseminated intravascular coagulation, leading to platelet depletion and severe hemorrhage. The viral protein p72 suppresses the expression of coagulation factor F10 both *in vivo* and *in vitro*. KAP1 enhances this suppressive effect of p72 on F10, thereby exacerbating disruption of the coagulation cascade and contributing to ASFV-induced pathological damage (Zhu et al., 2024).

To provide a virus-centric overview of KAP1’s diverse antiviral roles described above, we summarize the major viral systems and their corresponding regulatory mechanisms in **Table 3**.

**Table 3:** Functional roles of KAP1 in viral infection.

Virus	KAP1 mechanism	KAP1 function	Regulatory outcome
ERVs	Transcriptional repression	KAP1 recruits SETDB1 and HP1 to silence ERVs.	Suppresses viral transcription and replication
HIV-1	Transcriptional repression	KAP1 promotes CDK9 SUMOylation, blocks P-TEFb assembly, and cooperates with CTIP2 to degrade Tat.	Suppresses viral transcription and replication
	Relief of transcriptional repression	KAP1 is phosphorylated at Ser824 by DNA-PK through its multi-domain structure.	Enhances viral replication
MMLV	Transcriptional repression	KAP1 interacts with ZFP809 to repress pre-viral transcription, involving YY1, histone H3.3, and K799 in ESCs.	Suppresses viral gene expression
	SUMOylation/Ubiquitination	KAP1 degrades ZFP809 in differentiated cells, lifting transcriptional repression.	Promotes viral gene expression
PFV	Transcriptional repression	KAP1 induces histone methylation at the LTR promoter and mediates Tas degradation.	Suppresses viral replication
	SUMOylation/Ubiquitination	KAP1 mediates Tas ubiquitination and promotes proteasomal degradation.	Suppresses viral protein function
EBV	Transcriptional repression	KAP1 is SUMOylated by CTAR3, cooperates with IFI16 to silence EBV, inhibits BZLF1, and regulates BBLF2/3	Suppresses viral transcription and replication
	Relief of transcriptional repression	KAP1 is phosphorylated at Ser824 by BZLF1, chloroquine, and TXNIP, relieving transcriptional repression.	Enhances viral replication
KSHV	Transcriptional repression	KAP1 is SUMOylated by LANA with NRF2 involvement.	Suppresses viral transcription and replication
	Relief of transcriptional repression	KAP1 is regulated by STAT3, de-SUMOylated by HIF-1α, phosphorylated by Kaposin B, and its ubiquitination-mediated degradation of TFIIB is disrupted by KSHV	Promotes viral transcription
HCMV	Transcriptional repression	KAP1 recruits SERBP1 to silence the early promoter MIEP.	Suppresses viral gene expression
	Relief of transcriptional repression	KAP1 is phosphorylated at Ser824 by mTOR and chloroquine.	Enhances viral replication
HSV-1	Relief of transcriptional repression	KAP1 is phosphorylated at Ser824 by ILK, eliminating its repressive function.	Enhances viral replication
SARS-CoV-2	SUMOylation/Ubiquitination	KAP1 catalyzes SUMOylation of SARS2-NP to suppress innate immune signaling.	Promotes viral immune evasion
	Innate immune modulation	KAP1 modulates IFN/HERV pathways and acts as a previral factor.	Promotes immune evasion and pathogenesis
	Host receptor regulation	KAP1 inhibits ACE2 protein expression.	Restricts viral entry
MCPyV	Relief of transcriptional repression	KAP1 phosphorylation eliminates its transcriptional repression activity.	Enhances viral transcription
HBV	SUMOylation/Ubiquitination	KAP1 ubiquitinates and degrades HBx.	Suppresses viral replication
PRRSV	SUMOylation/Ubiquitination	KAP1 promotes K63-linked ubiquitination of GP4, enhancing viral protein expression.	Enhances viral replication or particle production
IAV	Innate immune modulation	KAP1 de-SUMOylation derepresses ERVs, triggering innate immunity.	Activates host antiviral response
HPAIV	Relief of transcriptional repression	KAP1 is phosphorylated at Ser473, activating MAPK signaling and pro-inflammatory gene expression.	Activates host innate immune response
RSV	Innate immune modulation	KAP1 expression correlates with IFN-mediated antiviral response.	Activates host innate immune signaling
PEDV	Viral immune evasion	KAP1 is hijacked by PEDVN to inhibit the JAK-STAT1 pathway.	Facilitates viral immune escape
ASFV	Virus-induced pathology	KAP1 inhibits ASFV-induced coagulation cascades, aggravating tissue damage.	Contributes to virus-induced pathology
HPV	Transcriptional repression	KAP1 acts as a co-repressor of E2F and represses E6/E7 expression through chromatin remodeling	KAP1 may facilitate HPV persistence and oncogenesis
HCV	Relief of transcriptional repression	KAP1 suppresses HERVs but is functionally impaired during HCV infection	Facilitates HCV replication and viral particle aggregation
HAdVs	Relief of transcriptional repression	KAP1 is de-SUMOylated after infection but enhances SUMOylation of E1B-55K	Enhances viral replication

## 5 Discussion

KAP1 is a multifaceted regulatory protein composed of distinct structural and functional domains, with its physiological roles widely studied in cellular and animal models. Central to its function is KRAB-ZFP-mediated gene silencing and the dynamic regulation of heterochromatin and euchromatin. SUMOylation enhances its transcriptional repression activity and plays a key role in maintaining genomic integrity (Cheng, 2014; Santoni De Sio, 2014; Ecco et al., 2017). Recent discoveries of novel KAP1-interacting transcription factors have further clarified its role in chromatin remodeling and transcriptional control (Bacon et al., 2020; Zhang et al., 2023b). In immune-related diseases such as multiple sclerosis (MS), KAP1 repression is subject to fine-tuned regulation, underscoring its immunomodulatory potential (Tovo et al., 2022, 2023c; Vianelli et al., 2022). Functional loss of KAP1 leads to broad phenotypic consequences, including impaired embryonic development and defective stem cell differentiation (Cammas et al., 2000; Sampath Kumar et al., 2017).

KAP1 has emerged as a central regulator of viral gene silencing, particularly in the context of endogenous retroviruses (ERVs). Through the recruitment of SETDB1 and HP1, KAP1 facilitates the establishment of heterochromatin at ERV loci, thereby repressing their transcription. SUMOylation of KAP1 further promotes its localization to ERV regions and stabilizes its interaction with SETDB1. Additional layers of suppression involve DNA methylation and RNA-mediated targeting of transposable elements, reinforcing KAP1-dependent ERV silencing (Yang et al., 2015; Brattås et al., 2017; Margalit et al., 2020).

In contrast, the role of KAP1 in HIV-1 regulation remains mechanistically complex and context-dependent. Accumulating evidence indicates that KAP1 functions as both a transcriptional repressor and activator, likely modulated by post-translational modifications (PTMs) in response to viral or host-derived signals. During latency, KAP1 reinforces transcriptional silencing through SUMOylation and ubiquitination of P-TEFb and Tat, with ZNF304 facilitating its recruitment to the HIV-1 promoter. Conversely, phosphorylation at Ser824 by DNA-PK converts KAP1 into a transcriptional activator, promoting viral reactivation (Taura et al., 2019; Ait-Ammar et al., 2021). These findings highlight KAP1 as a tunable molecular switch, whose activity may be selectively manipulated by targeting specific PTM sites or upstream kinases. Modulating this switch could inform future “shock-and-kill” strategies for HIV-1 eradication. By contrast, in other retroviruses such as MMLV and PFV, KAP1 appears to operate in a more canonical repressive manner, although further investigation is warranted (Lee et al., 2018; Bren et al., 2024; Sharma et al., 2024).

Herpesviruses, in particular, seem to have evolved precise mechanisms to counteract KAP1-mediated silencing. KAP1 initially represses herpesviral lytic genes via heterochromatinization, but during lytic reactivation, the virus hijacks cellular and viral kinases to phosphorylate KAP1 at Ser824, thereby reversing its repressor function. This dynamic suggests that herpesviruses efficiently exploit the KAP1 “switch”. Whether Ser824 phosphorylation can serve as a biomarker for predicting reactivation risk or as a therapeutic target remains to be determined *in vivo* (Bhaduri-McIntosh and Rousseau, 2024).

Beyond retroviruses and herpesviruses, recent studies have uncovered novel KAP1 functions in other viral systems. For instance, during SARS-CoV-2 infection, KAP1 expression levels correlate positively with disease severity and interferon (IFN) production (Tovo et al., 2021; Pellegrina et al., 2022; Tavakoli et al., 2022). Similar associations have been observed in IAV infection, particularly with HPAIV strains (Krischuns et al., 2018; Hale, 2022). These observations suggest that viral infection may trigger de-repression of KAP1-regulated endogenous retroviral (ERV) elements, leading to the accumulation of self-derived ERV RNAs that are sensed by the innate immune system. The resulting activation of IFN responses may amplify inflammatory signaling and contribute to severe immunopathology, including the so-called “cytokine storm” (Krischuns et al., 2018).

Phosphorylation at distinct sites further exemplifies how KAP1 integrates diverse cellular signals. Ser824 phosphorylation is predominantly induced by ATM kinase in response to DNA double-strand breaks, facilitating chromatin relaxation and DNA repair (Liu et al., 2015). By contrast, Ser473 phosphorylation is triggered by virus-activated cascades such as PKR-MAPK-MSK1, disrupting KAP1-HP1 interactions and enhancing inflammatory gene expression (Krischuns et al., 2018). These modifications illustrate how KAP1 functions as a molecular switch, balancing genome stability and antiviral defense.

Apart from its inhibitory role in transcriptional regulation, KAP1 also functions as a SUMO and E3 ubiquitin ligase through its RBCC domain, regulating not only viral proteins but also the post-translational modifications (PTMs) of key components in the host antiviral signaling pathways. Interestingly, KAP1 mediates the ubiquitination and SUMOylation of MAVS and IRF7, targeting them for degradation and thereby disrupting innate immune signaling and facilitating viral replication (Liang et al., 2011). In contrast, KAP1 enhances the stability of RLRs and TBK1, promoting IFN-mediated antiviral responses (Hua et al., 2024; Yang et al., 2024). The differential regulation of these signaling proteins by KAP1 may reflect virus-specific strategies or differences in experimental systems, warranting further investigation.

Given KAP1’s broad physiological roles, a single virus may elicit divergent—and at times opposing—regulatory outcomes. For example, KAP1 promotes the ubiquitination and degradation of the HBx protein, while simultaneously suppressing the HBV-induced antiviral immune response-two effects that together facilitate viral replication (Liu et al., 2023a; Yang et al., 2025). In the case of SARS-CoV-2, KAP1 not only represses viral transcription but also SUMOylates the nucleocapsid protein (SARS2-NP) and regulates ACE2 receptor expression, thereby influencing viral replication through multiple pathways (Tovo et al., 2021; Wang et al., 2021; Ren et al., 2024). Experimental heterogeneity may further contribute to conflicting observations. KAP1 represses MMLV in embryonic stem cells but enhances chromatin relaxation and viral proliferation in differentiated cells—a mechanistic paradox yet to be resolved (Elsässer et al., 2015; Wang and Goff, 2017; Lee et al., 2018). Likewise, studies on HIV-1 have yielded inconsistent findings depending on the knockdown approach. These discrepancies emphasize the importance of context-specific and *in vivo* investigations to fully elucidate KAP1’s regulatory versatility in viral infection.

**Figure 4:**
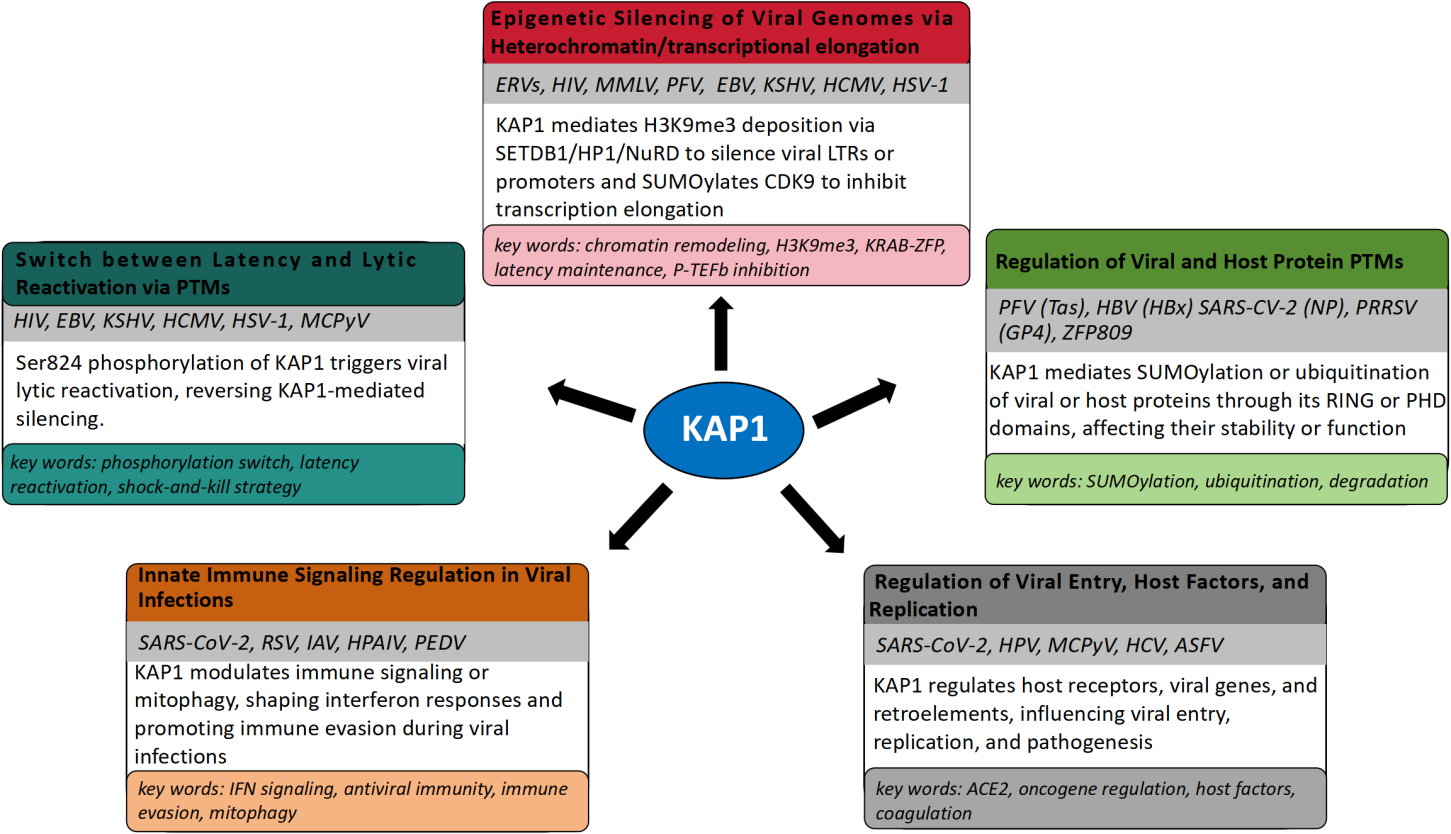
Modular framework illustrating the diverse mechanisms by which KAP1 regulates viral infection and host responses. Five functional modules summarize KAP1’s roles in epigenetic silencing, latency-lytic control, post-translational modification of viral and host proteins, modulation of innate immunity, and regulation of viral entry and pathogenicity. Representative viruses involved in each module are indicated.

To integrate the diverse regulatory roles described above, we categorized KAP1’s functions across viral systems into five mechanistic modules (**Figure 4**). This framework underscores its multifaceted involvement in viral silencing, immune modulation, and host-pathogen interactions.

In summary, KAP1 serves as a versatile master regulator that orchestrates diverse cellular and viral processes. Its ability to integrate chromatin remodeling, transcriptional repression, post-translational modifications, and immune regulation highlights its central role in host-virus dynamics. Moving forward, future research should adopt standardized experimental systems and incorporate *in vivo* models to delineate the molecular logic by which KAP1 toggles between antiviral defense and viral facilitation. Elucidating these mechanisms will be essential for understanding how KAP1 maintains genomic and immune homeostasis and for developing targeted strategies to modulate its activity in disease contexts.

## References

[B1] Acharya A. Ambikan A. T. Thurman M. Malik M. R. Dyavar S. R. Végvári Á.. (2023). Proteomic landscape of astrocytes and pericytes infected with HIV/SARS-CoV-2 mono/co-infection, impacting on neurological complications. rs.3.rs-3031591. doi: 10.21203/rs.3.rs-3031591/v1, PMID: 37398206 PMC10312942

[B2] Ait-Ammar A. Bellefroid M. Daouad F. Martinelli V. Van Assche J. Wallet C.. (2021). Inhibition of HIV-1 gene transcription by KAP1 in myeloid lineage. Sci. Rep. 11, 2692. doi: 10.1038/s41598-021-82164-w, PMID: 33514850 PMC7846785

[B3] Ak A. K. Bhutta B. S. Mendez M. D. (2025). “Herpes simplex encephalitis,” in StatPearls (StatPearls Publishing, Treasure Island (FL). Available online at: http://www.ncbi.nlm.nih.gov/books/NBK557643/. 32491575

[B4] Ali S. R. Jordan M. Nagarajan P. Amit M. (2022). Nerve density and neuronal biomarkers in cancer. Cancers 14, 4817. doi: 10.3390/cancers14194817, PMID: 36230740 PMC9561962

[B5] Allouch A. Di Primio C. Alpi E. Lusic M. Arosio D. Giacca M.. (2011). The TRIM family protein KAP1 inhibits HIV-1 integration. Cell Host Microbe 9, 484–495. doi: 10.1016/j.chom.2011.05.004, PMID: 21669397

[B6] Ashraf U. M. Abokor A. A. Edwards J. M. Waigi E. W. Royfman R. S. Hasan S. A.-M.. (2021). SARS-CoV-2, ACE2 expression, and systemic organ invasion. Physiol. Genomics 53, 51–60. doi: 10.1152/physiolgenomics.00087.2020, PMID: 33275540 PMC7900915

[B7] Asimi V. Sampath Kumar A. Niskanen H. Riemenschneider C. Hetzel S. Naderi J.. (2022). Hijacking of transcriptional condensates by endogenous retroviruses. Nat. Genet. 54, 1238–1247. doi: 10.1038/s41588-022-01132-w, PMID: 35864192 PMC9355880

[B8] Bacon C. W. Challa A. Hyder U. Shukla A. Borkar A. N. Bayo J.. (2020). KAP1 is a chromatin reader that couples steps of RNA polymerase II transcription to sustain oncogenic programs. Mol. Cell 78, 1133–1151.e14. doi: 10.1016/j.molcel.2020.04.024, PMID: 32402252 PMC7305985

[B9] Ballmer D. Tardat M. Ortiz R. Graff-Meyer A. Ozonov E. A. Genoud C.. (2023). HP1 proteins regulate nucleolar structure and function by secluding pericentromeric constitutive heterochromatin. Nucleic Acids Res. 51, 117–143. doi: 10.1093/nar/gkac1159, PMID: 36533441 PMC9841413

[B10] Barde I. Rauwel B. Marin-Florez R. M. Corsinotti A. Laurenti E. Verp S.. (2013). A KRAB/KAP1-miRNA cascade regulates erythropoiesis through stage-specific control of mitophagy. Science 340, 350–353. doi: 10.1126/science.1232398, PMID: 23493425 PMC3678075

[B11] Bentz G. L. Moss C. R. Whitehurst C. B. Moody C. A. Pagano J. S. (2015). LMP1-induced sumoylation influences the maintenance of epstein-barr virus latency through KAP1. J. Virol. 89, 7465–7477. doi: 10.1128/JVI.00711-15, PMID: 25948750 PMC4505653

[B12] Bhaduri-McIntosh S. Rousseau B. A. (2024). KAP1/TRIM28 – antiviral and proviral protagonist of herpesvirus biology. Trends Microbiol. 32, 1179–1189. doi: 10.1016/j.tim.2024.05.007, PMID: 38871562 PMC11620967

[B13] Bhatia N. Xiao T. Z. Rosenthal K. A. Siddiqui I. A. Thiyagarajan S. Smart B.. (2013). MAGE-C2 promotes growth and tumorigenicity of melanoma cells, phosphorylation of KAP1, and DNA damage repair. J. Invest. Dermatol. 133, 759–767. doi: 10.1038/jid.2012.355, PMID: 23096706 PMC3570725

[B14] Bojkowska K. Aloisio F. Cassano M. Kapopoulou A. De Sio F. S. Zangger N.. (2012). Liver-specific ablation of Krüppel-associated box–associated protein 1 in mice leads to male-predominant hepatosteatosis and development of liver adenoma. Hepatology 56, 1279–1290. doi: 10.1002/hep.25767, PMID: 22684873 PMC4894457

[B15] Bolderson E. Savage K. I. Mahen R. Pisupati V. Graham M. E. Richard D. J.. (2012). Krüppel-associated box (KRAB)-associated co-repressor (KAP-1) ser-473 phosphorylation regulates heterochromatin protein 1β (HP1-β) mobilization and DNA repair in heterochromatin. J. Biol. Chem. 287, 28122–28131. doi: 10.1074/jbc.M112.368381, PMID: 22715096 PMC3431694

[B16] Bove R. Sutton P. Nicholas J. (2024). Women’s health and pregnancy in multiple sclerosis. Neurologic Clinics 42, 275–293. doi: 10.1016/j.ncl.2023.07.004, PMID: 37980119

[B17] Brattås P. L. Jönsson M. E. Fasching L. Nelander Wahlestedt J. Shahsavani M. Falk R.. (2017). TRIM28 controls a gene regulatory network based on endogenous retroviruses in human neural progenitor cells. Cell Rep. 18, 1–11. doi: 10.1016/j.celrep.2016.12.010, PMID: 28052240

[B18] Bren I. Tal A. Strauss C. Schlesinger S. (2024). The role of Smarcad1 in retroviral repression in mouse embryonic stem cells. Mobile DNA 15, 4. doi: 10.1186/s13100-024-00314-z, PMID: 38468276 PMC10929159

[B19] Broussard G. Damania B. (2020). Regulation of KSHV latency and lytic reactivation. Viruses 12, 1034. doi: 10.3390/v12091034, PMID: 32957532 PMC7551196

[B20] Bunch H. Calderwood S. K. (2015). TRIM28 as a novel transcriptional elongation factor. BMC Mol. Biol. 16, 14. doi: 10.1186/s12867-015-0040-x, PMID: 26293668 PMC4545989

[B21] Bürck C. Mund A. Berscheminski J. Kieweg L. Müncheberg S. Dobner T.. (2016). KAP1 is a host restriction factor that promotes human adenovirus E1B-55K SUMO modification. J. Virol. 90, 930–946. doi: 10.1128/JVI.01836-15, PMID: 26537675 PMC4702688

[B22] Burton E. M. Akinyemi I. A. Frey T. R. Xu H. Li X. Su L. J.. (2021). A heterochromatin inducing protein differentially recognizes self versus foreign genomes. PloS Pathog. 17, e1009447. doi: 10.1371/journal.ppat.1009447, PMID: 33730092 PMC8007004

[B23] Burton E. M. Goldbach-Mansky R. Bhaduri-McIntosh S. (2020). A promiscuous inflammasome sparks replication of a common tumor virus. Proc. Natl. Acad. Sci. U.S.A. 117, 1722–1730. doi: 10.1073/pnas.1919133117, PMID: 31919284 PMC6983388

[B24] Cammas F. Mark M. Dollé P. Dierich A. Chambon P. Losson R. (2000). Mice lacking the transcriptional corepressor TIF1β are defective in early postimplantation development. Development 127, 2955–2963. doi: 10.1242/dev.127.13.2955, PMID: 10851139

[B25] Cao X. Chen Y. Chen Y. Jiang M. (2024). The role of tripartite motif family proteins in chronic liver diseases: molecular mechanisms and therapeutic potential. Biomolecules 14, 1038. doi: 10.3390/biom14081038, PMID: 39199424 PMC11352684

[B26] Carusillo A. Mussolino C. (2020). DNA damage: from threat to treatment. Cells 9, 1665. doi: 10.3390/cells9071665, PMID: 32664329 PMC7408370

[B27] Chang C.-W. Chou H.-Y. Lin Y.-S. Huang K.-H. Chang C.-J. Hsu T.-C.. (2008). Phosphorylation at Ser473 regulates heterochromatin protein 1 binding and corepressor function of TIF1beta/KAP1. BMC Mol. Biol. 9, 61. doi: 10.1186/1471-2199-9-61, PMID: 18590578 PMC2474647

[B28] Chang P.-C. Fitzgerald L. D. Van Geelen A. Izumiya Y. Ellison T. J. Wang D.-H.. (2009). Kruppel-associated box domain-associated protein-1 as a latency regulator for kaposi’s sarcoma-associated herpesvirus and its modulation by the viral protein kinase. Cancer Res. 69, 5681–5689. doi: 10.1158/0008-5472.CAN-08-4570, PMID: 19584288 PMC2731626

[B29] Chang J. Hwang H. J. Kim B. Choi Y.-G. Park J. Park Y.. (2021). TRIM28 functions as a negative regulator of aggresome formation. Autophagy 17, 4231–4248. doi: 10.1080/15548627.2021.1909835, PMID: 33783327 PMC8726693

[B30] Chelmicki T. Roger E. Teissandier A. Dura M. Bonneville L. Rucli S.. (2021). m6A RNA methylation regulates the fate of endogenous retroviruses. Nature 591, 312–316. doi: 10.1038/s41586-020-03135-1, PMID: 33442060

[B31] Chen Y.-Y. Ran X.-H. Ni R.-Z. Mu D. (2023). TRIM28 negatively regulates the RLR signaling pathway by targeting MAVS for degradation via K48-linked polyubiquitination. J. Biol. Chem. 299, 104660. doi: 10.1016/j.jbc.2023.104660, PMID: 37119745 PMC10165269

[B32] Cheng C.-T. (2014). KAPtain in charge of multiple missions: Emerging roles of KAP1. WJBC 5, 308. doi: 10.4331/wjbc.v5.i3.308, PMID: 25225599 PMC4160525

[B33] Chikuma S. Suita N. Okazaki I.-M. Shibayama S. Honjo T. (2012). TRIM28 prevents autoinflammatory T cell development in *vivo* . Nat. Immunol. 13, 596–603. doi: 10.1038/ni.2293, PMID: 22544392

[B34] Chikuma S. Yamanaka S. Nakagawa S. Ueda M. T. Hayabuchi H. Tokifuji Y.. (2021). TRIM28 expression on dendritic cells prevents excessive T cell priming by silencing endogenous retrovirus. J. Immunol. 206, 1528–1539. doi: 10.4049/jimmunol.2001003, PMID: 33619215

[B35] Chou T. C. Maggirwar N. S. Marsden M. D. (2024). HIV persistence, latency, and cure approaches: where are we now? Viruses 16, 1163. doi: 10.3390/v16071163, PMID: 39066325 PMC11281696

[B36] Cohen J. I. (2020). Herpesvirus latency. J. Clin. Invest. 130, 3361–3369. doi: 10.1172/JCI136225, PMID: 32364538 PMC7324166

[B37] Crawford K. Lager K. M. Kulshreshtha V. Miller L. C. Faaberg K. S. (2016). Status of vaccines for porcine epidemic diarrhea virus in the United States and Canada. Virus Res. 226, 108–116. doi: 10.1016/j.virusres.2016.08.005, PMID: 27545066

[B38] Cui Z. Zhou L. Zhao S. Li W. Li J. Chen J.. (2023). The host E3-ubiquitin ligase TRIM28 impedes viral protein GP4 ubiquitination and promotes PRRSV replication. IJMS 24, 10965. doi: 10.3390/ijms241310965, PMID: 37446143 PMC10341522

[B39] Czerwińska P. Mazurek S. Wiznerowicz M. (2017). The complexity of TRIM28 contribution to cancer. J. BioMed. Sci. 24, 63. doi: 10.1186/s12929-017-0374-4, PMID: 28851455 PMC5574234

[B40] D’Orso I. (2016). 7SKiing on chromatin: Move globally, act locally. RNA Biol. 13, 545–553. doi: 10.1080/15476286.2016.1181254, PMID: 27128603 PMC4962805

[B41] Da Costa I. C. Schmidt C. K. (2020). Ubiquitin-like proteins in the DNA damage response: the next generation. Essays Biochem. 64, 737–752. doi: 10.1042/EBC20190095, PMID: 32451552

[B42] Damania B. Kenney S. C. Raab-Traub N. (2022). Epstein-Barr virus: Biology and clinical disease. Cell 185, 3652–3670. doi: 10.1016/j.cell.2022.08.026, PMID: 36113467 PMC9529843

[B43] De La Cruz-Herrera C. F. Tatham M. H. Siddiqi U. Z. Shire K. Marcon E. Greenblatt J. F.. (2023). Changes in SUMO-modified proteins in Epstein-Barr virus infection identifies reciprocal regulation of TRIM24/28/33 complexes and the lytic switch BZLF1. PloS Pathog. 19, e1011477. doi: 10.1371/journal.ppat.1011477, PMID: 37410772 PMC10353822

[B44] Dobson R. Giovannoni G. (2019). Multiple sclerosis – a review. Euro J. Neurol. 26, 27–40. doi: 10.1111/ene.13819, PMID: 30300457

[B45] Ecco G. Imbeault M. Trono D. (2017). KRAB zinc finger proteins. Development 144, 2719–2729. doi: 10.1242/dev.132605, PMID: 28765213 PMC7117961

[B46] Elsässer S. J. Noh K.-M. Diaz N. Allis C. D. Banaszynski L. A. (2015). Histone H3.3 is required for endogenous retroviral element silencing in embryonic stem cells. Nature 522, 240–244. doi: 10.1038/nature14345, PMID: 25938714 PMC4509593

[B47] Enriquez-Gasca R. Gould P. A. Tunbak H. Conde L. Herrero J. Chittka A.. (2023). Co-option of endogenous retroviruses through genetic escape from TRIM28 repression. Cell Rep. 42, 112625. doi: 10.1016/j.celrep.2023.112625, PMID: 37294634 PMC11980785

[B48] Fasching L. Kapopoulou A. Sachdeva R. Petri R. Jönsson M. E. Männe C.. (2015). TRIM28 represses transcription of endogenous retroviruses in neural progenitor cells. Cell Rep. 10, 20–28. doi: 10.1016/j.celrep.2014.12.004, PMID: 25543143 PMC4434221

[B49] Feng H. Yi R. Wu S. Wang G. Sun R. Lin L.. (2022). KAP1 positively modulates influenza A virus replication by interacting with PB2 and NS1 proteins in human lung epithelial cells. Viruses 14, 689. doi: 10.3390/v14040689, PMID: 35458419 PMC9025026

[B50] Fonti G. Marcaida M. J. Bryan L. C. Träger S. Kalantzi A. S. Helleboid P.-Y. J.. (2019). KAP1 is an antiparallel dimer with a functional asymmetry. Life Sci. Alliance 2, e201900349. doi: 10.26508/lsa.201900349, PMID: 31427381 PMC6701479

[B51] Forte E. Swaminathan S. Schroeder M. W. Kim J. Y. Terhune S. S. Hummel M. (2018). Tumor necrosis factor alpha induces reactivation of human cytomegalovirus independently of myeloid cell differentiation following posttranscriptional establishment of latency. mBio 9, e01560–e01518. doi: 10.1128/mBio.01560-18, PMID: 30206173 PMC6134100

[B52] Friedman J. R. Fredericks W. J. Jensen D. E. Speicher D. W. Huang X. P. Neilson E. G.. (1996). KAP-1, a novel corepressor for the highly conserved KRAB repression domain. Genes Dev. 10, 2067–2078. doi: 10.1101/gad.10.16.2067, PMID: 8769649

[B53] Fukuda K. Shinkai Y. (2020). SETDB1-mediated silencing of retroelements. Viruses 12, 596. doi: 10.3390/v12060596, PMID: 32486217 PMC7354471

[B54] Gan J. Wang C. Jin Y. Guo Y. Xu F. Zhu Q.. (2015). Proteomic profiling identifies the SIM-associated complex of KSHV-encoded LANA. Proteomics 15, 2023–2037. doi: 10.1002/pmic.201400624, PMID: 25894481 PMC5868752

[B55] Gao X. Li Q. Chen G. He H. Ma Y. (2020). MAGEA3 promotes proliferation and suppresses apoptosis in cervical cancer cells by inhibiting the KAP1/p53 signaling pathway. Am. J. Transl. Res. 12, 3596–3612., PMID: 32774721 PMC7407682

[B56] Gao F. Wen G. (2025). Strategies and scheming: the war between PRRSV and host cells. Virol. J. 22, 191. doi: 10.1186/s12985-025-02685-y, PMID: 40500743 PMC12153163

[B57] Gareau J. R. Lima C. D. (2010). The SUMO pathway: emerging mechanisms that shape specificity, conjugation and recognition. Nat. Rev. Mol. Cell Biol. 11, 861–871. doi: 10.1038/nrm3011, PMID: 21102611 PMC3079294

[B58] Garigliany M. Van Laere A.-S. Clercx C. Giet D. Escriou N. Huon C.. (2020). SARS-CoV-2 natural transmission from human to cat, Belgium, march 2020. Emerg. Infect. Dis. 26, 3069–3071. doi: 10.3201/eid2612.202223, PMID: 32788033 PMC7706966

[B59] Geis F. K. Goff S. P. (2020). Silencing and transcriptional regulation of endogenous retroviruses: an overview. Viruses 12, 884. doi: 10.3390/v12080884, PMID: 32823517 PMC7472088

[B60] Germini D. Sall F. B. Shmakova A. Wiels J. Dokudovskaya S. Drouet E.. (2020). Oncogenic properties of the EBV ZEBRA protein. Cancers (Basel) 12, 1479. doi: 10.3390/cancers12061479, PMID: 32517128 PMC7352903

[B61] Gjyshi O. Roy A. Dutta S. Veettil M. V. Dutta D. Chandran B. (2015). Activated nrf2 interacts with kaposi’s sarcoma-associated herpesvirus latency protein LANA-1 and host protein KAP1 to mediate global lytic gene repression. J. Virol. 89, 7874–7892. doi: 10.1128/JVI.00895-15, PMID: 25995248 PMC4505678

[B62] Goodarzi A. A. Kurka T. Jeggo P. A. (2011). KAP-1 phosphorylation regulates CHD3 nucleosome remodeling during the DNA double-strand break response. Nat. Struct. Mol. Biol. 18, 831–839. doi: 10.1038/nsmb.2077, PMID: 21642969

[B63] Greenwood A. D. Ishida Y. O’Brien S. P. Roca A. L. Eiden M. V. (2018). Transmission, evolution, and endogenization: lessons learned from recent retroviral invasions. Microbiol. Mol. Biol. Rev. 82, e00044–e00017. doi: 10.1128/MMBR.00044-17, PMID: 29237726 PMC5813887

[B64] Grewal S. I. S. (2023). The molecular basis of heterochromatin assembly and epigenetic inheritance. Mol. Cell 83, 1767–1785. doi: 10.1016/j.molcel.2023.04.020, PMID: 37207657 PMC10309086

[B65] Griffiths P. Reeves M. (2021). Pathogenesis of human cytomegalovirus in the immunocompromised host. Nat. Rev. Microbiol. 19, 759–773. doi: 10.1038/s41579-021-00582-z, PMID: 34168328 PMC8223196

[B66] Gugliesi F. Pasquero S. Griffante G. Scutera S. Albano C. Pacheco S. F. C.. (2021). Human cytomegalovirus and autoimmune diseases: where are we? Viruses 13, 260. doi: 10.3390/v13020260, PMID: 33567734 PMC7914970

[B67] Haggerty C. Kretzmer H. Riemenschneider C. Kumar A. S. Mattei A. L. Bailly N.. (2021). Dnmt1 has *de novo* activity targeted to transposable elements. Nat. Struct. Mol. Biol. 28, 594–603. doi: 10.1038/s41594-021-00603-8, PMID: 34140676 PMC8279952

[B68] Hale B. G. (2022). Antiviral immunity triggered by infection-induced host transposable elements. Curr. Opin. Virol. 52, 211–216. doi: 10.1016/j.coviro.2021.12.006, PMID: 34959082

[B69] Harms P. W. Harms K. L. Moore P. S. DeCaprio J. A. Nghiem P. Wong M. K. K.. (2018). The biology and treatment of Merkel cell carcinoma: current understanding and research priorities. Nat. Rev. Clin. Oncol. 15, 763–776. doi: 10.1038/s41571-018-0103-2, PMID: 30287935 PMC6319370

[B70] Harrison K. S. Jones C. (2022). Regulation of herpes simplex virus type 1 latency-reactivation cycle and ocular disease by cellular signaling pathways. Exp. Eye Res. 218, 109017. doi: 10.1016/j.exer.2022.109017, PMID: 35240194 PMC9191828

[B71] Hosoya T. Clifford M. Losson R. Tanabe O. Engel J. D. (2013). TRIM28 is essential for erythroblast differentiation in the mouse. Blood 122, 3798–3807. doi: 10.1182/blood-2013-04-496166, PMID: 24092935 PMC3843238

[B72] Hu C. Zhang S. Gao X. Gao X. Xu X. Lv Y.. (2012). Roles of kruppel-associated box (KRAB)-associated co-repressor KAP1 ser-473 phosphorylation in DNA damage response. J. Biol. Chem. 287, 18937–18952. doi: 10.1074/jbc.M111.313262, PMID: 22496453 PMC3365928

[B73] Hua F. Nass T. Parvatiyar K. (2024). TRIM28 facilitates type I interferon activation by targeting TBK1. Front. Immunol. 15. doi: 10.3389/fimmu.2024.1279920, PMID: 38495890 PMC10940511

[B74] Huang R.-X. Zhou P.-K. (2020). DNA damage response signaling pathways and targets for radiotherapy sensitization in cancer. Sig Transduct Target Ther. 5, 60. doi: 10.1038/s41392-020-0150-x, PMID: 32355263 PMC7192953

[B75] Hughes S. H. (2015). Reverse transcription of retroviruses and LTR retrotransposons. Microbiol. Spectr. 3, 3.2.18. doi: 10.1128/microbiolspec.MDNA3-0027-2014, PMID: 26104704 PMC6775776

[B76] Ivanov A. V. Peng H. Yurchenko V. Yap K. L. Negorev D. G. Schultz D. C.. (2007). PHD domain-mediated E3 ligase activity directs intramolecular sumoylation of an adjacent bromodomain required for gene silencing. Mol. Cell 28, 823–837. doi: 10.1016/j.molcel.2007.11.012, PMID: 18082607 PMC4348069

[B77] Iyengar S. Farnham P. J. (2011). KAP1 protein: an enigmatic master regulator of the genome. J. Biol. Chem. 286, 26267–26276. doi: 10.1074/jbc.R111.252569, PMID: 21652716 PMC3143589

[B78] Iyengar S. Ivanov A. V. Jin V. X. Rauscher F. J. Farnham P. J. (2011). Functional analysis of KAP1 genomic recruitment. Mol. Cell. Biol. 31, 1833–1847. doi: 10.1128/MCB.01331-10, PMID: 21343339 PMC3133220

[B79] Jackson C. B. Farzan M. Chen B. Choe H. (2022). Mechanisms of SARS-CoV-2 entry into cells. Nat. Rev. Mol. Cell Biol. 23, 3–20. doi: 10.1038/s41580-021-00418-x, PMID: 34611326 PMC8491763

[B80] Jakobsson J. Cordero M. I. Bisaz R. Groner A. C. Busskamp V. Bensadoun J.-C.. (2008). KAP1-mediated epigenetic repression in the forebrain modulates behavioral vulnerability to stress. Neuron 60, 818–831. doi: 10.1016/j.neuron.2008.09.036, PMID: 19081377

[B81] Jang S.-H. Choi H.-W. Ahn J. Jang S. Yoon J.-H. Lee M.-G.. (2024). XAF1 antagonizes TRIM28 activity through the assembly of a ZNF313-mediated destruction complex to suppress tumor Malignancy. Mol. BioMed. 5, 58. doi: 10.1186/s43556-024-00224-9, PMID: 39532800 PMC11557793

[B82] Jovčevska I. Zupanec N. Urlep Ž. Vranič A. Matos B. Stokin C. L.. (2017). Differentially expressed proteins in glioblastoma multiforme identified with a nanobody-based anti-proteome approach and confirmed by OncoFinder as possible tumor-class predictive biomarker candidates. Oncotarget 8, 44141–44158. doi: 10.18632/oncotarget.17390, PMID: 28498803 PMC5546469

[B83] Jung K. Saif L. J. Wang Q. (2020). Porcine epidemic diarrhea virus (PEDV): An update on etiology, transmission, pathogenesis, and prevention and control. Virus Res. 286, 198045. doi: 10.1016/j.virusres.2020.198045, PMID: 32502552 PMC7266596

[B84] Kajon A. E. (2024). Adenovirus infections: new insights for the clinical laboratory. J. Clin. Microbiol. 62, e0083622. doi: 10.1128/jcm.00836-22, PMID: 39189703 PMC11389149

[B85] Kanda T. (2018). “EBV-encoded latent genes,” in Human Herpesviruses. Eds. Kawaguchi Y. Mori Y. Kimura H. (Springer Singapore, Singapore), 377–394. doi: 10.1007/978-981-10-7230-7_17, PMID: 29896676

[B86] Kawabe H. Stegmüller J. (2021). The role of E3 ubiquitin ligases in synapse function in the healthy and diseased brain. Mol. Cell. Neurosci. 112, 103602. doi: 10.1016/j.mcn.2021.103602, PMID: 33581237

[B87] Khetchoumian K. Teletin M. Mark M. Lerouge T. Cerviño M. Oulad-Abdelghani M.. (2004). TIF1δ, a novel HP1-interacting member of the transcriptional intermediary factor 1 (TIF1) family expressed by elongating spermatids. J. Biol. Chem. 279, 48329–48341. doi: 10.1074/jbc.M404779200, PMID: 15322135

[B88] Kim S.-S. Chen Y.-M. O’Leary E. Witzgall R. Vidal M. Bonventre J. V. (1996). A novel member of the RING finger family, KRIP-1, associates with the KRAB-A transcriptional repressor domain of zinc finger proteins. Proc. Natl. Acad. Sci. U.S.A. 93, 15299–15304. doi: 10.1073/pnas.93.26.15299, PMID: 8986806 PMC26399

[B89] Kimura Y. Nagao A. Fujioka Y. Satou A. Taira T. Iguchi-Ariga S. M. M.. (2007). MM-1 facilitates degradation of c-Myc by recruiting proteasome and a novel ubiquitin E3 ligase. Int. J. Oncol. 31, 829–836. doi: 10.3892/ijo.31.4.829, PMID: 17786314

[B90] King C. A. (2013). Kaposi’s sarcoma-associated herpesvirus kaposin B induces unique monophosphorylation of STAT3 at serine 727 and MK2-mediated inactivation of the STAT3 transcriptional repressor TRIM28. J. Virol. 87, 8779–8791. doi: 10.1128/JVI.02976-12, PMID: 23740979 PMC3719813

[B91] King C. A. Li X. Barbachano-Guerrero A. Bhaduri-McIntosh S. (2015). STAT3 regulates lytic activation of kaposi’s sarcoma-associated herpesvirus. J. Virol. 89, 11347–11355. doi: 10.1128/JVI.02008-15, PMID: 26339061 PMC4645641

[B92] Köcher S. Zech H. B. Krug L. Gatzemeier F. Christiansen S. Meyer F.. (2022). A lack of effectiveness in the ATM-orchestrated DNA damage response contributes to the DNA repair defect of HPV-positive head and neck cancer cells. Front. Oncol. 12. doi: 10.3389/fonc.2022.765968, PMID: 35719921 PMC9204973

[B93] Kotobuki Y. Tonomura K. Fujimoto M. (2021). Transcriptional intermediary factor 1 (TIF1) and anti-TIF1γ antibody-positive dermatomyositis. Immunol. Med. 44, 23–29. doi: 10.1080/25785826.2020.1791402, PMID: 32649853

[B94] Krasnopolsky S. Kuzmina A. Taube R. (2020). Genome-wide CRISPR knockout screen identifies ZNF304 as a silencer of HIV transcription that promotes viral latency. PloS Pathog. 16, e1008834. doi: 10.1371/journal.ppat.1008834, PMID: 32956422 PMC7529202

[B95] Krebs A.-S. Mendonça L. M. Zhang P. (2021). Structural analysis of retrovirus assembly and maturation. Viruses 14, 54. doi: 10.3390/v14010054, PMID: 35062258 PMC8778513

[B96] Krischuns T. Günl F. Henschel L. Binder M. Willemsen J. Schloer S.. (2018). Phosphorylation of TRIM28 enhances the expression of IFN-β and proinflammatory cytokines during HPAIV infection of human lung epithelial cells. Front. Immunol. 9. doi: 10.3389/fimmu.2018.02229, PMID: 30323812 PMC6172303

[B97] Kuang M. Zhao Y. Yu H. Li S. Liu T. Chen L.. (2023). XAF1 promotes anti-RNA virus immune responses by regulating chromatin accessibility. Sci. Adv. 9, eadg5211. doi: 10.1126/sciadv.adg5211, PMID: 37595039 PMC10438455

[B98] Kuo C.-Y. Li X. Kong X.-Q. Luo C. Chang C.-C. Chung Y.. (2014). An arginine-rich motif of ring finger protein 4 (RNF4) oversees the recruitment and degradation of the phosphorylated and SUMOylated krüppel-associated box domain-associated protein 1 (KAP1)/TRIM28 protein during genotoxic stress. J. Biol. Chem. 289, 20757–20772. doi: 10.1074/jbc.M114.555672, PMID: 24907272 PMC4110285

[B99] Kuo C.-Y. Li X. Stark J. M. Shih H.-M. Ann D. K. (2016). RNF4 regulates DNA double-strand break repair in a cell cycle-dependent manner. Cell Cycle 15, 787–798. doi: 10.1080/15384101.2016.1138184, PMID: 26766492 PMC4845925

[B100] Lechner M. S. Begg G. E. Speicher D. W. Rauscher F. J. (2000). Molecular determinants for targeting heterochromatin protein 1-mediated gene silencing: direct chromoshadow domain–KAP-1 corepressor interaction is essential. Mol. Cell. Biol. 20, 6449–6465. doi: 10.1128/MCB.20.17.6449-6465.2000, PMID: 10938122 PMC86120

[B101] Lee A. CingÖz O. Sabo Y. Goff S. P. (2018). Characterization of interaction between Trim28 and YY1 in silencing proviral DNA of Moloney murine leukemia virus. Virology 516, 165–175. doi: 10.1016/j.virol.2018.01.012, PMID: 29407374 PMC8456507

[B102] Lee D.-H. Goodarzi A. A. Adelmant G. O. Pan Y. Jeggo P. A. Marto J. A.. (2012). Phosphoproteomic analysis reveals that PP4 dephosphorylates KAP-1 impacting the DNA damage response: PP4 regulates KAP-1 function in DDR. EMBO J. 31, 2403–2415. doi: 10.1038/emboj.2012.86, PMID: 22491012 PMC3364739

[B103] Lee Y.-K. Thomas S. N. Yang A. J. Ann D. K. (2007). Doxorubicin down-regulates krüppel-associated box domain-associated protein 1 sumoylation that relieves its transcription repression on p21WAF1/CIP1 in breast cancer MCF-7 cells. J. Biol. Chem. 282, 1595–1606. doi: 10.1074/jbc.M606306200, PMID: 17079232

[B104] Leonardi L. Rivalta B. Leone F. Cancrini C. Caffarelli C. Marseglia G. L.. (2022). Host defenses to viruses: lessons from inborn errors of immunity. Medicina 58, 248. doi: 10.3390/medicina58020248, PMID: 35208572 PMC8879264

[B105] Li X. Burton E. M. Bhaduri-McIntosh S. (2017). Chloroquine triggers Epstein-Barr virus replication through phosphorylation of KAP1/TRIM28 in Burkitt lymphoma cells. PloS Pathog. 13, e1006249. doi: 10.1371/journal.ppat.1006249, PMID: 28249048 PMC5348047

[B106] Li X. Burton E. M. Koganti S. Zhi J. Doyle F. Tenenbaum S. A.. (2018). KRAB-ZFP repressors enforce quiescence of oncogenic human herpesviruses. J. Virol. 92, e00298–e00218. doi: 10.1128/JVI.00298-18, PMID: 29695433 PMC6026741

[B107] Li H. Chen M. Zheng T. Lei X. Lin C. Li S.. (2024a). IFITM1 and IFITM2 inhibit the replication of senecavirus A by positive feedback with RIG-I signaling pathway. Veterinary Microbiol. 292, 110050. doi: 10.1016/j.vetmic.2024.110050, PMID: 38484578

[B108] Li J. Cheng H. Zhao Y. Wang Y. Gong C. Gong R.. (2024b). ZNF331 represses the proliferation of head and neck squamous cell carcinoma via co-repressor TRIM28 . Oral. Dis. 31, odi.15209. doi: 10.1111/odi.15209, PMID: 39587824

[B109] Li X. Kozlov S. V. El-Guindy A. Bhaduri-McIntosh S. (2019). Retrograde regulation by the viral protein kinase epigenetically sustains the epstein-barr virus latency-to-lytic switch to augment virus production. J. Virol. 93, e00572–e00519. doi: 10.1128/JVI.00572-19, PMID: 31189703 PMC6694827

[B110] Li X. Lin H. H. Chen H. Xu X. Shih H.-M. Ann D. K. (2010). SUMOylation of the transcriptional co-repressor KAP1 is regulated by the serine and threonine phosphatase PP1. Sci. Signal. 3, ra32. doi: 10.1126/scisignal.2000781, PMID: 20424263 PMC3302164

[B111] Li Q. Qin Y. Wang W. Jia M. Zhao W. Zhao C. (2021). KAP1-mediated epigenetic suppression in anti-RNA viral responses by direct targeting RIG-I and MDA5. J. Immunol. 207, 1903–1910. doi: 10.4049/jimmunol.2100342, PMID: 34497149

[B112] Li M. Xu X. Chang C.-W. Liu Y. (2020). TRIM28 functions as the SUMO E3 ligase for PCNA in prevention of transcription induced DNA breaks. Proc. Natl. Acad. Sci. U.S.A. 117, 23588–23596. doi: 10.1073/pnas.2004122117, PMID: 32900933 PMC7519263

[B113] Li X. Yan Z. Ma J. Li G. Liu X. Peng Z.. (2024c). TRIM28 promotes porcine epidemic diarrhea virus replication by mitophagy-mediated inhibition of the JAK-STAT1 pathway. Int. J. Biol. Macromolecules 254, 127722. doi: 10.1016/j.ijbiomac.2023.127722, PMID: 37907173

[B114] Liang Q. Deng H. Li X. Wu X. Tang Q. Chang T.-H.. (2011). Tripartite motif-containing protein 28 is a small ubiquitin-related modifier E3 ligase and negative regulator of IFN regulatory factor 7. J. Immunol. 187, 4754–4763. doi: 10.4049/jimmunol.1101704, PMID: 21940674 PMC3197880

[B115] Lin J. Guo D. Liu H. Zhou W. Wang C. Müller I.. (2021). The SETDB1–TRIM28 complex suppresses antitumor immunity. Cancer Immunol. Res. 9, 1413–1424. doi: 10.1158/2326-6066.CIR-21-0754, PMID: 34848497 PMC8647838

[B116] Lin Y.-H. Yuan J. Pei H. Liu T. Ann D. K. Lou Z. (2015). KAP1 deacetylation by SIRT1 promotes non-homologous end-joining repair. PloS One 10, e0123935. doi: 10.1371/journal.pone.0123935, PMID: 25905708 PMC4408008

[B117] Linney E. Davis B. Overhauser J. Chao E. Fan H. (1984). Non-function of a Moloney murine leukaemia virus regulatory sequence in F9 embryonal carcinoma cells. Nature 308, 470–472. doi: 10.1038/308470a0, PMID: 6323996

[B118] Lion T. (2014). Adenovirus infections in immunocompetent and immunocompromised patients. Clin. Microbiol. Rev. 27, 441–462. doi: 10.1128/CMR.00116-13, PMID: 24982316 PMC4135893

[B119] Liu S. Cai X. Wu J. Cong Q. Chen X. Li T.. (2015). Phosphorylation of innate immune adaptor proteins MAVS, STING, and TRIF induces IRF3 activation. Science 347, aaa2630. doi: 10.1126/science.aaa2630, PMID: 25636800

[B120] Liu Y. Cao B. Hu L. Ye J. Tian W. He X. (2022). The dual roles of MAGE-C2 in p53 ubiquitination and cell proliferation through E3 ligases MDM2 and TRIM28. Front. Cell Dev. Biol. 10. doi: 10.3389/fcell.2022.922675, PMID: 35927984 PMC9344466

[B121] Liu H. Chen H. Deng X. Peng Y. Zeng Q. Song Z.. (2019). Knockdown of TRIM28 inhibits PDGF-BB-induced vascular smooth muscle cell proliferation and migration. Chemico-Biological Interact. 311, 108772. doi: 10.1016/j.cbi.2019.108772, PMID: 31351049

[B122] Liu X. Gan J. Du S. Zhu C. Wang Y. Jia Y.. (2021b). Proteomic profiling identifies kaposi’s sarcoma-associated herpesvirus (KSHV)-encoded LANA^SIM^ -associated proteins in hypoxia. mSystems 6, e01109–e01121. doi: 10.1128/mSystems.01109-21, PMID: 34726485 PMC8562486

[B123] Liu J. Gao M. He J. Wu K. Lin S. Jin L.. (2021a). The RNA m6A reader YTHDC1 silences retrotransposons and guards ES cell identity. Nature 591, 322–326. doi: 10.1038/s41586-021-03313-9, PMID: 33658714

[B124] Liu H.-L. Nan H. Zhao W.-W. Wan X.-B. Fan X.-J. (2024). Phase separation in DNA double-strand break response. Nucleus 15, 2296243. doi: 10.1080/19491034.2023.2296243, PMID: 38146123 PMC10761171

[B125] Liu C. Zhao K. Chen Y. Yao Y. Tang J. Wang J.. (2023a). Mitochondrial glycerol-3-phosphate dehydrogenase restricts HBV replication via the TRIM28-mediated degradation of HBx. J. Virol. 97, e00580–e00523. doi: 10.1128/jvi.00580-23, PMID: 37166302 PMC10231258

[B126] Liu W. Zhu Y. Ye W. Xiong J. Wang H. Gao Y.. (2025). Redox regulation of TRIM28 facilitates neuronal ferroptosis by promoting SUMOylation and inhibiting OPTN-selective autophagic degradation of ACSL4. Cell Death Differ 32, 1041–1057. doi: 10.1038/s41418-025-01452-4, PMID: 39875520 PMC12162872

[B127] Liu F. Zhuang W. Song B. Yang Y. Liu J. Zheng Y.. (2023b). MAVS-loaded unanchored Lys63-linked polyubiquitin chains activate the RIG-I-MAVS signaling cascade. Cell Mol. Immunol. 20, 1186–1202. doi: 10.1038/s41423-023-01065-2, PMID: 37582970 PMC10542333

[B128] Lobanova Y. Filonova G. Kaplun D. Zhigalova N. Prokhortchouk E. Zhenilo S. (2023). TRIM28 regulates transcriptional activity of methyl-DNA binding protein Kaiso by SUMOylation. Biochimie 206, 73–80. doi: 10.1016/j.biochi.2022.10.006, PMID: 36252888

[B129] Lork M. Lieber G. Hale B. G. (2021). Proteomic approaches to dissect host SUMOylation during innate antiviral immune responses. Viruses 13, 528. doi: 10.3390/v13030528, PMID: 33806893 PMC8004987

[B130] Lu R. Zhao X. Li J. Niu P. Yang B. Wu H.. (2020). Genomic characterisation and epidemiology of 2019 novel coronavirus: implications for virus origins and receptor binding. Lancet 395, 565–574. doi: 10.1016/S0140-6736(20)30251-8, PMID: 32007145 PMC7159086

[B131] Luo J. Zhang Y. Guo Y. Tang H. Wei H. Liu S.. (2017). TRIM28 regulates Igf2-H19 and Dlk1-Gtl2 imprinting by distinct mechanisms during sheep fibroblast proliferation. Gene 637, 152–160. doi: 10.1016/j.gene.2017.09.048, PMID: 28947302

[B132] Ma X. Yang T. Luo Y. Wu L. Jiang Y. Song Z.. (2019). TRIM28 promotes HIV-1 latency by SUMOylating CDK9 and inhibiting P-TEFb. eLife 8, e42426. doi: 10.7554/eLife.42426, PMID: 30652970 PMC6361614

[B133] Margalit L. Strauss C. Tal A. Schlesinger S. (2020). Trim24 and trim33 play a role in epigenetic silencing of retroviruses in embryonic stem cells. Viruses 12, 1015. doi: 10.3390/v12091015, PMID: 32932986 PMC7551373

[B134] McAvera R. M. Crawford L. J. (2020). TIF1 proteins in genome stability and cancer. Cancers 12, 2094. doi: 10.3390/cancers12082094, PMID: 32731534 PMC7463590

[B135] McNamara R. P. Guzman C. Reeder J. E. D’Orso I. (2016a). Genome-wide analysis of KAP1, the 7SK snRNP complex, and RNA polymerase II. Genomics Data 7, 250–255. doi: 10.1016/j.gdata.2016.01.019, PMID: 26981421 PMC4778668

[B136] McNamara R. P. Reeder J. E. McMillan E. A. Bacon C. W. McCann J. L. D’Orso I. (2016b). KAP1 recruitment of the 7SK snRNP complex to promoters enables transcription elongation by RNA polymerase II. Mol. Cell 61, 39–53. doi: 10.1016/j.molcel.2015.11.004, PMID: 26725010 PMC4714561

[B137] Meroni G. Diez-Roux G. (2005). TRIM/RBCC, a novel class of ‘single protein RING finger’ E3 ubiquitin ligases. BioEssays 27, 1147–1157. doi: 10.1002/bies.20304, PMID: 16237670

[B138] Messerschmidt D. M. De Vries W. Ito M. Solter D. Ferguson-Smith A. Knowles B. B. (2012). *Trim28* is required for epigenetic stability during mouse oocyte to embryo transition. Science 335, 1499–1502. doi: 10.1126/science.1216154, PMID: 22442485

[B139] Metzger M. B. Pruneda J. N. Klevit R. E. Weissman A. M. (2014). RING-type E3 ligases: Master manipulators of E2 ubiquitin-conjugating enzymes and ubiquitination. Biochim. Biophys. Acta (BBA) - Mol. Cell Res. 1843, 47–60. doi: 10.1016/j.bbamcr.2013.05.026, PMID: 23747565 PMC4109693

[B140] Miyazato P. Matsuo M. Katsuya H. Satou Y. (2016). Transcriptional and epigenetic regulatory mechanisms affecting HTLV-1 provirus. Viruses 8, 171. doi: 10.3390/v8060171, PMID: 27322309 PMC4926191

[B141] Morii M. Kubota S. Iimori M. Yokomizo-Nakano T. Hamashima A. Bai J.. (2024). TIF1β activates leukemic transcriptional program in HSCs and promotes BCR::ABL1-induced myeloid leukemia. Leukemia 38, 1275–1286. doi: 10.1038/s41375-024-02276-w, PMID: 38734786

[B142] Morton E. L. Forst C. V. Zheng Y. DePaula-Silva A. B. Ramirez N.-G. P. Planelles V.. (2019). Transcriptional circuit fragility influences HIV proviral fate. Cell Rep. 27, 154–171.e9. doi: 10.1016/j.celrep.2019.03.007, PMID: 30943398 PMC6461408

[B143] Nyenhuis D. A. Watanabe S. M. Tjandra N. Carter C. A. (2025). Tsg101 mimicry of canonical E2 enzymes underlies its role in ubiquitin signaling. Proc. Natl. Acad. Sci. U.S.A. 122, e2419542121. doi: 10.1073/pnas.2419542121, PMID: 39739800 PMC11725782

[B144] Oksenych V. Kainov D. E. (2021). DNA damage response. Biomolecules 11, 123. doi: 10.3390/biom11010123, PMID: 33477863 PMC7832852

[B145] Ouyang C. Lu G. He W. Bay B.-H. Shen H.-M. (2022). Post-translational modification in control of SIRT1 stability during DNA damage response. Int. J. Biol. Sci. 18, 2655–2669. doi: 10.7150/ijbs.68587, PMID: 35541916 PMC9066097

[B146] Padeken J. Methot S. P. Gasser S. M. (2022). Establishment of H3K9-methylated heterochromatin and its functions in tissue differentiation and maintenance. Nat. Rev. Mol. Cell Biol. 23, 623–640. doi: 10.1038/s41580-022-00483-w, PMID: 35562425 PMC9099300

[B147] Park H.-H. Kim H.-R. Park S.-Y. Hwang S.-M. Hong S. M. Park S.. (2021). RIPK3 activation induces TRIM28 derepression in cancer cells and enhances the anti-tumor microenvironment. Mol. Cancer 20, 107. doi: 10.1186/s12943-021-01399-3, PMID: 34419074 PMC8379748

[B148] Pavlaki I. Alammari F. Sun B. Clark N. Sirey T. Lee S.. (2018). The long non-coding RNA *Paupar* promotes KAP 1-dependent chromatin changes and regulates olfactory bulb neurogenesis. EMBO J. 37, e98219. doi: 10.15252/embj.201798219, PMID: 29661885 PMC5978383

[B149] Pellegrina D. Bahcheli A. T. Krassowski M. Reimand J. (2022). Human phospho-signaling networks of SARS-CoV-2 infection are rewired by population genetic variants. Mol. Syst. Biol. 18, e10823. doi: 10.15252/msb.202110823, PMID: 35579274 PMC9112486

[B150] Peng H. Begg G. E. Schultz D. C. Friedman J. R. Jensen D. E. Speicher D. W.. (2000). Reconstitution of the KRAB-KAP-1 repressor complex: a model system for defining the molecular anatomy of RING-B box-coiled-coil domain-mediated protein-protein interactions. J. Mol. Biol. 295, 1139–1162. doi: 10.1006/jmbi.1999.3402, PMID: 10653693

[B151] Peng H. Gibson L. C. Capili A. D. Borden K. L. B. Osborne M. J. Harper S. L.. (2007). The Structurally Disordered KRAB Repression Domain Is Incorporated into a Protease Resistant Core upon Binding to KAP-1-RBCC Domain. J. Mol. Biol. 370, 269–289. doi: 10.1016/j.jmb.2007.03.047, PMID: 17512541

[B152] Peng J. Wysocka J. (2008). It takes a PHD to SUMO. Trends Biochem. Sci. 33, 191–194. doi: 10.1016/j.tibs.2008.02.003, PMID: 18406149

[B153] Pisano G. Roy A. Ahmed Ansari M. Kumar B. Chikoti L. Chandran B. (2017). Interferon-γ-inducible protein 16 (IFI16) is required for the maintenance of Epstein-Barr virus latency. Virol. J. 14, 221. doi: 10.1186/s12985-017-0891-5, PMID: 29132393 PMC5683537

[B154] Poole E. Sinclair J. (2022). Latency-associated upregulation of SERBP1 is important for the recruitment of transcriptional repressors to the viral major immediate early promoter of human cytomegalovirus during latent carriage. Front. Microbiol. 13. doi: 10.3389/fmicb.2022.999290, PMID: 36504797 PMC9729347

[B155] Qin Y. Li Q. Liang W. Yan R. Tong L. Jia M.. (2021). TRIM28 SUMOylates and stabilizes NLRP3 to facilitate inflammasome activation. Nat. Commun. 12, 4794. doi: 10.1038/s41467-021-25033-4, PMID: 34373456 PMC8352945

[B156] Randolph K. Hyder U. Challa A. Perez E. D’Orso I. (2024). Functional analysis of KAP1/TRIM28 requirements for HIV-1 transcription activation. Viruses 16, 116. doi: 10.3390/v16010116, PMID: 38257816 PMC10819576

[B157] Randolph K. Hyder U. D’Orso I. (2022). KAP1/TRIM28: transcriptional activator and/or repressor of viral and cellular programs? Front. Cell. Infect. Microbiol. 12. doi: 10.3389/fcimb.2022.834636, PMID: 35281453 PMC8904932

[B158] Rapone R. Del Maestro L. Bouyioukos C. Albini S. Cruz-Tapias P. Joliot V.. (2023). The cytoplasmic fraction of the histone lysine methyltransferase Setdb1 is essential for embryonic stem cells. iScience 26, 107386. doi: 10.1016/j.isci.2023.107386, PMID: 37559904 PMC10407132

[B159] Rauwel B. Jang S. M. Cassano M. Kapopoulou A. Barde I. Trono D. (2015). Release of human cytomegalovirus from latency by a KAP1/TRIM28 phosphorylation switch. eLife 4, e06068. doi: 10.7554/eLife.06068, PMID: 25846574 PMC4384640

[B160] Reichel A. Stilp A.-C. Scherer M. Reuter N. Lukassen S. Kasmapour B.. (2018). Chromatin-remodeling factor SPOC1 acts as a cellular restriction factor against human cytomegalovirus by repressing the major immediate early promoter. J. Virol. 92, e00342–e00318. doi: 10.1128/JVI.00342-18, PMID: 29743358 PMC6026729

[B161] Ren J. Wang S. Zong Z. Pan T. Liu S. Mao W.. (2024). TRIM28-mediated nucleocapsid protein SUMOylation enhances SARS-CoV-2 virulence. Nat. Commun. 15, 244. doi: 10.1038/s41467-023-44502-6, PMID: 38172120 PMC10764958

[B162] Robbez-Masson L. Tie C. H. C. Conde L. Tunbak H. Husovsky C. Tchasovnikarova I. A.. (2018). The HUSH complex cooperates with TRIM28 to repress young retrotransposons and new genes. Genome Res. 28, 836–845. doi: 10.1101/gr.228171.117, PMID: 29728366 PMC5991525

[B163] Rosspopoff O. Trono D. (2023). Take a walk on the KRAB side. Trends Genet. 39, 844–857. doi: 10.1016/j.tig.2023.08.003, PMID: 37716846

[B164] Rowe H. M. Jakobsson J. Mesnard D. Rougemont J. Reynard S. Aktas T.. (2010). KAP1 controls endogenous retroviruses in embryonic stem cells. Nature 463, 237–240. doi: 10.1038/nature08674, PMID: 20075919

[B165] Rozman B. Nachshon A. Levi Samia R. Lavi M. Schwartz M. Stern-Ginossar N. (2022). Temporal dynamics of HCMV gene expression in lytic and latent infections. Cell Rep. 39, 110653. doi: 10.1016/j.celrep.2022.110653, PMID: 35417700 PMC9035752

[B166] Sahu R. K. Dhakshnamoorthy J. Jain S. Folco H. D. Wheeler D. Grewal S. I. S. (2024). Nucleosome remodeler exclusion by histone deacetylation enforces heterochromatic silencing and epigenetic inheritance. Mol. Cell 84, 3175–3191.e8. doi: 10.1016/j.molcel.2024.07.006, PMID: 39096900 PMC11649001

[B167] Sakai M. Masuda Y. Tarumoto Y. Aihara N. Tsunoda Y. Iwata M.. (2024). Genome-scale CRISPR-Cas9 screen identifies host factors as potential therapeutic targets for SARS-CoV-2 infection. iScience 27, 110475. doi: 10.1016/j.isci.2024.110475, PMID: 39100693 PMC11295705

[B168] Sales-Gil R. Vagnarelli P. (2020). How HP1 post-translational modifications regulate heterochromatin formation and maintenance. Cells 9, 1460. doi: 10.3390/cells9061460, PMID: 32545538 PMC7349378

[B169] Sampath Kumar A. Seah M. K. Y. Ling K. Y. Wang Y. Tan J. H. L. Nitsch S.. (2017). Loss of maternal *Trim28* causes male-predominant early embryonic lethality. Genes Dev. 31, 12–17. doi: 10.1101/gad.291195.116, PMID: 28115466 PMC5287108

[B170] Santoni De Sio F. R. (2014). Kruppel-associated box (KRAB) proteins in the adaptive immune system. Nucleus 5, 138–148. doi: 10.4161/nucl.28738, PMID: 24699165 PMC4049920

[B171] Santoni De Sio F. R. Massacand J. Barde I. Offner S. Corsinotti A. Kapopoulou A.. (2012). KAP1 regulates gene networks controlling mouse B-lymphoid cell differentiation and function. Blood 119, 4675–4685. doi: 10.1182/blood-2011-12-401117, PMID: 22452978 PMC3683646

[B172] Santos J. Gil J. (2014). TRIM28/KAP1 regulates senescence. Immunol. Lett. 162, 281–289. doi: 10.1016/j.imlet.2014.08.011, PMID: 25160591

[B173] Schichl K. Doorbar J. (2025). Regulation and deregulation of viral gene expression during high-risk HPV infection. Viruses 17, 937. doi: 10.3390/v17070937, PMID: 40733555 PMC12299310

[B174] Schmidt N. Domingues P. Golebiowski F. Patzina C. Tatham M. H. Hay R. T.. (2019). An influenza virus-triggered SUMO switch orchestrates co-opted endogenous retroviruses to stimulate host antiviral immunity. Proc. Natl. Acad. Sci. U.S.A. 116, 17399–17408. doi: 10.1073/pnas.1907031116, PMID: 31391303 PMC6717285

[B175] Schneeberger P. E. Bierhals T. Neu A. Hempel M. Kutsche K. (2019). *de novo* MEPCE nonsense variant associated with a neurodevelopmental disorder causes disintegration of 7SK snRNP and enhanced RNA polymerase II activation. Sci. Rep. 9, 12516. doi: 10.1038/s41598-019-49032-0, PMID: 31467394 PMC6715695

[B176] Schoelz J. M. Riddle N. C. (2022). Functions of HP1 proteins in transcriptional regulation. Epigenet. Chromatin 15, 14. doi: 10.1186/s13072-022-00453-8, PMID: 35526078 PMC9078007

[B177] Schultz D. C. Ayyanathan K. Negorev D. Maul G. G. Rauscher F. J. (2002). SETDB1: a novel KAP-1-associated histone H3, lysine 9-specific methyltransferase that contributes to HP1-mediated silencing of euchromatic genes by KRAB zinc-finger proteins. Genes Dev. 16, 919–932. doi: 10.1101/gad.973302, PMID: 11959841 PMC152359

[B178] Shah P. A. Boutros-Suleiman S. Emanuelli A. Paolini B. Levy-Cohen G. Blank M. (2022). The emerging role of E3 ubiquitin ligase SMURF2 in the regulation of transcriptional co-repressor KAP1 in untransformed and cancer cells and tissues. Cancers 14, 1607. doi: 10.3390/cancers14071607, PMID: 35406379 PMC8997158

[B179] Sharma A. L. Tyagi P. Khumallambam M. Tyagi M. (2024). Cocaine-induced DNA-dependent protein kinase relieves RNAP II pausing by promoting TRIM28 phosphorylation and RNAP II hyperphosphorylation to enhance HIV transcription. Cells 13, 1950. doi: 10.3390/cells13231950, PMID: 39682697 PMC11640508

[B180] Siebels S. Czech-Sioli M. Spohn M. Schmidt C. Theiss J. Indenbirken D.. (2020). Merkel cell polyomavirus DNA replication induces senescence in human dermal fibroblasts in a kap1/trim28-dependent manner. mBio 11, e00142–e00120. doi: 10.1128/mBio.00142-20, PMID: 32156811 PMC7064754

[B181] Sio F. R. S. Barde I. Offner S. Kapopoulou A. Corsinotti A. Bojkowska K.. (2012). KAP1 regulates gene networks controlling T-cell development and responsiveness. FASEB J. 26, 4561–4575. doi: 10.1096/fj.12-206177, PMID: 22872677 PMC4894473

[B182] Soldan S. S. Lieberman P. M. (2023). Epstein–Barr virus and multiple sclerosis. Nat. Rev. Microbiol. 21, 51–64. doi: 10.1038/s41579-022-00770-5, PMID: 35931816 PMC9362539

[B183] Spearman C. W. Dusheiko G. M. Hellard M. Sonderup M. (2019). Hepatitis C. Lancet 394, 1451–1466. doi: 10.1016/S0140-6736(19)32320-7, PMID: 31631857

[B184] Sripathy S. P. Stevens J. Schultz D. C. (2006). The KAP1 corepressor functions to coordinate the assembly of *de novo* HP1-demarcated microenvironments of heterochromatin required for KRAB zinc finger protein-mediated transcriptional repression. Mol. Cell. Biol. 26, 8623–8638. doi: 10.1128/MCB.00487-06, PMID: 16954381 PMC1636786

[B185] Steiner S. Kratzel A. Barut G. T. Lang R. M. Aguiar Moreira E. Thomann L.. (2024). SARS-CoV-2 biology and host interactions. Nat. Rev. Microbiol. 22, 206–225. doi: 10.1038/s41579-023-01003-z, PMID: 38225365

[B186] Stoll G. A. Oda S. Chong Z.-S. Yu M. McLaughlin S. H. Modis Y. (2019). Structure of KAP1 tripartite motif identifies molecular interfaces required for retroelement silencing. Proc. Natl. Acad. Sci. U.S.A. 116, 15042–15051. doi: 10.1073/pnas.1901318116, PMID: 31289231 PMC6660772

[B187] Stoll G. A. Pandiloski N. Douse C. H. Modis Y. (2022). Structure and functional mapping of the KRAB-KAP1 repressor complex. EMBO J. 41, e111179. doi: 10.15252/embj.2022111179, PMID: 36341546 PMC9753469

[B188] Sun Y. Keown J. R. Black M. M. Raclot C. Demarais N. Trono D.. (2019). A dissection of oligomerization by the TRIM28 tripartite motif and the interaction with members of the krab-ZFP family. J. Mol. Biol. 431, 2511–2527. doi: 10.1016/j.jmb.2019.05.002, PMID: 31078555

[B189] Sun R. Liang D. Gao Y. Lan K. (2014). Kaposi’s sarcoma-associated herpesvirus-encoded LANA interacts with host KAP1 to facilitate establishment of viral latency. J. Virol. 88, 7331–7344. doi: 10.1128/JVI.00596-14, PMID: 24741090 PMC4054432

[B190] Swinkels H. M. Nguyen A. D. Gulick P. G. (2025). “HIV and AIDS,” in StatPearls (StatPearls Publishing, Treasure Island (FL). Available online at: http://www.ncbi.nlm.nih.gov/books/NBK534860/.

[B191] Taka J. R. H. Sun Y. Goldstone D. C. (2022). Mapping the interaction between Trim28 and the KRAB domain at the center of Trim28 silencing of endogenous retroviruses. Protein Sci. 31, e4436. doi: 10.1002/pro.4436, PMID: 36173157 PMC9601868

[B192] Tan J. Sun X. Zhao H. Guan H. Gao S. Zhou P. (2023). Double-strand DNA break repair: molecular mechanisms and therapeutic targets. MedComm 4, e388. doi: 10.1002/mco2.388, PMID: 37808268 PMC10556206

[B193] Tanaka S. Pfleger C. Lai J.-F. Roan F. Sun S.-C. Ziegler S. F. (2018). KAP1 regulates regulatory T cell function and proliferation in both foxp3-dependent and -independent manners. Cell Rep. 23, 796–807. doi: 10.1016/j.celrep.2018.03.099, PMID: 29669285 PMC5947873

[B194] Taura M. Song E. Ho Y.-C. Iwasaki A. (2019). Apobec3A maintains HIV-1 latency through recruitment of epigenetic silencing machinery to the long terminal repeat. Proc. Natl. Acad. Sci. U.S.A. 116, 2282–2289. doi: 10.1073/pnas.1819386116, PMID: 30670656 PMC6369738

[B195] Tavakoli R. Rahimi P. Hamidi-Fard M. Eybpoosh S. Doroud D. Ahmadi I.. (2022). Comparing the expression levels of tripartite motif containing 28 in mild and severe COVID-19 infection. Virol. J. 19, 156. doi: 10.1186/s12985-022-01885-0, PMID: 36192760 PMC9527726

[B196] Thierry E. Brennich M. Round A. Buisson M. Burmeister W. P. Hutin S. (2015). Production and characterisation of Epstein–Barr virus helicase–primase complex and its accessory protein BBLF2/3. Virus Genes 51, 171–181. doi: 10.1007/s11262-015-1233-6, PMID: 26292944

[B197] Thiru A. Nietlispach D. Mott H. R. Okuwaki M. Lyon D. Nielsen P. R.. (2004). Structural basis of HP1/PXVXL motif peptide interactions and HP1 localisation to heterochromatin. EMBO J. 23, 489–499. doi: 10.1038/sj.emboj.7600088, PMID: 14765118 PMC1271814

[B198] Tie C. H. Fernandes L. Conde L. Robbez-Masson L. Sumner R. P. Peacock T.. (2018). KAP 1 regulates endogenous retroviruses in adult human cells and contributes to innate immune control. EMBO Rep. 19, e45000. doi: 10.15252/embr.201745000, PMID: 30061100 PMC6172469

[B199] Tovo P.-A. Davico C. Marcotulli D. Vitiello B. Daprà V. Calvi C.. (2022). Enhanced expression of human endogenous retroviruses, TRIM28 and SETDB1 in autism spectrum disorder. IJMS 23, 5964. doi: 10.3390/ijms23115964, PMID: 35682642 PMC9180946

[B200] Tovo P.-A. Galliano I. Parodi E. Calvi C. Gambarino S. Licciardi F.. (2023a). Children with chronic immune thrombocytopenia exhibit high expression of human endogenous retroviruses TRIM28 and SETDB1. Genes 14, 1569. doi: 10.3390/genes14081569, PMID: 37628621 PMC10454145

[B201] Tovo P.-A. Garazzino S. Daprà V. Alliaudi C. Silvestro E. Calvi C.. (2020a). Chronic HCV infection is associated with overexpression of human endogenous retroviruses that persists after drug-induced viral clearance. IJMS 21, 3980. doi: 10.3390/ijms21113980, PMID: 32492928 PMC7313012

[B202] Tovo P.-A. Garazzino S. Daprà V. Pruccoli G. Calvi C. Mignone F.. (2021). COVID-19 in children: expressions of type I/II/III interferons, TRIM28, SETDB1, and endogenous retroviruses in mild and severe cases. IJMS 22, 7481. doi: 10.3390/ijms22147481, PMID: 34299101 PMC8303145

[B203] Tovo P.-A. Garazzino S. Savino F. Daprà V. Pruccoli G. Dini M.. (2023b). Expressions of type I and III interferons, endogenous retroviruses, TRIM28, and SETDB1 in children with respiratory syncytial virus bronchiolitis. CIMB 45, 1197–1217. doi: 10.3390/cimb45020079, PMID: 36826024 PMC9954910

[B204] Tovo P.-A. Marozio L. Abbona G. Calvi C. Frezet F. Gambarino S.. (2023c). Pregnancy is associated with impaired transcription of human endogenous retroviruses and of TRIM28 and SETDB1, particularly in mothers affected by multiple sclerosis. Viruses 15, 710. doi: 10.3390/v15030710, PMID: 36992419 PMC10051116

[B205] Tovo P.-A. Rabbone I. Tinti D. Galliano I. Trada M. Daprà V.. (2020b). Enhanced expression of human endogenous retroviruses in new-onset type 1 diabetes: Potential pathogenetic and therapeutic implications. Autoimmunity 53, 283–288. doi: 10.1080/08916934.2020.1777281, PMID: 32586158

[B206] Tsai M.-S. Chen S.-H. Chang C.-P. Hsiao Y.-L. Wang L.-C. (2022). Integrin-linked kinase reduces H3K9 trimethylation to enhance herpes simplex virus 1 replication. Front. Cell. Infect. Microbiol. 12. doi: 10.3389/fcimb.2022.814307, PMID: 35350437 PMC8957879

[B207] Turelli P. Castro-Diaz N. Marzetta F. Kapopoulou A. Raclot C. Duc J.. (2014). Interplay of TRIM28 and DNA methylation in controlling human endogenous retroelements. Genome Res. 24, 1260–1270. doi: 10.1101/gr.172833.114, PMID: 24879559 PMC4120080

[B208] V’kovski P. Kratzel A. Steiner S. Stalder H. Thiel V. (2021). Coronavirus biology and replication: implications for SARS-CoV-2. Nat. Rev. Microbiol. 19, 155–170. doi: 10.1038/s41579-020-00468-6, PMID: 33116300 PMC7592455

[B209] Venturini L. You J. Stadler M. Galien R. Lallemand V. Koken M. H.. (1999). TIF1γ, a novel member of the transcriptional intermediary factor 1 family. Oncogene 18, 1209–1217. doi: 10.1038/sj.onc.1202655, PMID: 10022127

[B210] Vianelli N. Auteri G. Buccisano F. Carrai V. Baldacci E. Clissa C.. (2022). Refractory primary immune thrombocytopenia (ITP): current clinical challenges and therapeutic perspectives. Ann. Hematol. 101, 963–978. doi: 10.1007/s00277-022-04786-y, PMID: 35201417 PMC8867457

[B211] Volkmann E. R. Andréasson K. Smith V. (2023). Systemic sclerosis. Lancet 401, 304–318. doi: 10.1016/S0140-6736(22)01692-0, PMID: 36442487 PMC9892343

[B212] Wang Y. Du S. Zhu C. Wang C. Yu N. Lin Z.. (2020a). STUB1 is targeted by the SUMO-interacting motif of EBNA1 to maintain Epstein-Barr Virus latency. PloS Pathog. 16, e1008447. doi: 10.1371/journal.ppat.1008447, PMID: 32176739 PMC7105294

[B213] Wang Y. Fan Y. Huang Y. Du T. Liu Z. Huang D.. (2021). TRIM28 regulates SARS-CoV-2 cell entry by targeting ACE2. Cell. Signalling 85, 110064. doi: 10.1016/j.cellsig.2021.110064, PMID: 34146659 PMC8213541

[B214] Wang L. Gao Y. Zheng X. Liu C. Dong S. Li R.. (2019). Histone modifications regulate chromatin compartmentalization by contributing to a phase separation mechanism. Mol. Cell 76, 646–659.e6. doi: 10.1016/j.molcel.2019.08.019, PMID: 31543422

[B215] Wang C. Goff S. P. (2017). Differential control of retrovirus silencing in embryonic cells by proteasomal regulation of the ZFP809 retroviral repressor. Proc. Natl. Acad. Sci. U.S.A. 114, E922–E930. doi: 10.1073/pnas.1620879114, PMID: 28115710 PMC5307487

[B216] Wang C. Ivanov A. Chen L. Fredericks W. J. Seto E. Rauscher F. J.. (2005). MDM2 interaction with nuclear corepressor KAP1 contributes to p53 inactivation. EMBO J. 24, 3279–3290. doi: 10.1038/sj.emboj.7600791, PMID: 16107876 PMC1224681

[B217] Wang X. Li Y. Shi T. Bont L. J. Chu H. Y. Zar H. J.. (2024). Global disease burden of and risk factors for acute lower respiratory infections caused by respiratory syncytial virus in preterm infants and young children in 2019: a systematic review and meta-analysis of aggregated and individual participant data. Lancet 403, 1241–1253. doi: 10.1016/S0140-6736(24)00138-7, PMID: 38367641

[B218] Wang Y. Singh A. R. Zhao Y. Du T. Huang Y. Wan X.. (2020b). TRIM28 regulates sprouting angiogenesis through VEGFR-DLL4-Notch signaling circuit. FASEB J. 34, 14710–14724. doi: 10.1096/fj.202000186RRR, PMID: 32918765 PMC10115459

[B219] Wang G. Z. Wolf D. Goff S. P. (2014). EBP1, a novel host factor involved in primer binding site-dependent restriction of moloney murine leukemia virus in embryonic cells. J. Virol. 88, 1825–1829. doi: 10.1128/JVI.02578-13, PMID: 24227866 PMC3911593

[B220] Weber P. Cammas F. Gerard C. Metzger D. Chambon P. Losson R.. (2002). Germ cell expression of the transcriptional co-repressor TIF1β is required for the maintenance of spermatogenesis in the mouse. Development 129, 2329–2337. doi: 10.1242/dev.129.10.2329, PMID: 11973266

[B221] Weber M. Padmanabhan Nair V. Bauer T. Sprinzl M. F. Protzer U. Vincendeau M. (2021). Increased HERV-K(HML-2) transcript levels correlate with clinical parameters of liver damage in hepatitis C patients. Cells 10, 774. doi: 10.3390/cells10040774, PMID: 33807462 PMC8065411

[B222] Wei J. Sun Y. Wang T. Zhu G. Liu W. He X.. (2022). The regulation of prototype foamy virus 5′Long terminal repeats and internal promoter by endogenous transcription factors. Intervirology 65, 17–28. doi: 10.1159/000517539, PMID: 34438397 PMC8820438

[B223] White D. E. Negorev D. Peng H. Ivanov A. V. Maul G. G. Rauscher F. J. (2006). KAP1, a novel substrate for PIKK family members, colocalizes with numerous damage response factors at DNA lesions. Cancer Res. 66, 11594–11599. doi: 10.1158/0008-5472.CAN-06-4138, PMID: 17178852

[B224] White D. Rafalska-Metcalf I. U. Ivanov A. V. Corsinotti A. Peng H. Lee S.-C.. (2012). The ATM substrate KAP1 controls DNA repair in heterochromatin: regulation by HP1 proteins and serine 473/824 phosphorylation. Mol. Cancer Res. 10, 401–414. doi: 10.1158/1541-7786.MCR-11-0134, PMID: 22205726 PMC4894472

[B225] Wildenbeest J. G. Lowe D. M. Standing J. F. Butler C. C. (2024). Respiratory syncytial virus infections in adults: a narrative review. Lancet Respir. Med. 12, 822–836. doi: 10.1016/S2213-2600(24)00255-8, PMID: 39265602

[B226] Wolf D. Cammas F. Losson R. Goff S. P. (2008). Primer binding site-dependent restriction of murine leukemia virus requires HP1 binding by TRIM28. J. Virol. 82, 4675–4679. doi: 10.1128/JVI.02445-07, PMID: 18287239 PMC2293057

[B227] Wolf D. Goff S. P. (2007). TRIM28 mediates primer binding site-targeted silencing of murine leukemia virus in embryonic cells. Cell 131, 46–57. doi: 10.1016/j.cell.2007.07.026, PMID: 17923087

[B228] Xiao X. Fu Y. You W. Huang C. Zeng F. Gu X.. (2024). Inhibition of the RLR signaling pathway by SARS-CoV-2 ORF7b is mediated by MAVS and abrogated by ORF7b-homologous interfering peptide. J. Virol. 98, e01573–e01523. doi: 10.1128/jvi.01573-23, PMID: 38572974 PMC11092349

[B229] Xu H. Akinyemi I. A. Haley J. McIntosh M. T. Bhaduri-McIntosh S. (2023). ATM, KAP1 and the Epstein–Barr virus polymerase processivity factor direct traffic at the intersection of transcription and replication. Nucleic Acids Res. 51, 11104–11122. doi: 10.1093/nar/gkad823, PMID: 37852757 PMC10639065

[B230] Xu S. Jin T. Weng J. (2022b). Endothelial cells as a key cell type for innate immunity: A focused review on RIG-I signaling pathway. Front. Immunol. 13. doi: 10.3389/fimmu.2022.951614, PMID: 35865527 PMC9294349

[B231] Xu H. Li X. Rousseau B. A. Akinyemi I. A. Frey T. R. Zhou K.. (2022a). IFI16 partners with KAP1 to maintain epstein-barr virus latency. J. Virol. 96, e01028–e01022. doi: 10.1128/jvi.01028-22, PMID: 35969079 PMC9472614

[B232] Xue C. Meng H. Niu W. Li M. Wei J. Chen S.. (2024). TRIM28 promotes tumor growth and metastasis in breast cancer by targeting the BRD7 protein for ubiquitination and degradation. Cell Oncol. 47, 1973–1993. doi: 10.1007/s13402-024-00981-3, PMID: 39222175 PMC12974028

[B233] Yamauchi M. Freitag B. Khan C. Berwin B. Barklis E. (1995). Stem cell factor binding to retrovirus primer binding site silencers. J. Virol. 69, 1142–1149. doi: 10.1128/jvi.69.2.1142-1149.1995, PMID: 7529329 PMC188687

[B234] Yan Q. Zhou J. Gu Y. Huang W. Ruan M. Zhang H.. (2024). Lactylation of NAT10 promotes N4-acetylcytidine modification on tRNASer-CGA-1–1 to boost oncogenic DNA virus KSHV reactivation. Cell Death Differ 31, 1362–1374. doi: 10.1038/s41418-024-01327-0, PMID: 38879723 PMC11445560

[B235] Yang B. X. El Farran C. A. Guo H. C. Yu T. Fang H. T. Wang H. F.. (2015). Systematic identification of factors for provirus silencing in embryonic stem cells. Cell 163, 230–245. doi: 10.1016/j.cell.2015.08.037, PMID: 26365490 PMC4686136

[B236] Yang D. Geng T. Harrison A. G. Cahoon J. G. Xing J. Jiao B.. (2024). UBR5 promotes antiviral immunity by disengaging the transcriptional brake on RIG-I like receptors. Nat. Commun. 15, 780. doi: 10.1038/s41467-024-45141-1, PMID: 38278841 PMC10817939

[B237] Yang F. Tanasa B. Micheletti R. Ohgi K. A. Aggarwal A. K. Rosenfeld M. G. (2021). Shape of promoter antisense RNAs regulates ligand-induced transcription activation. Nature 595, 444–449. doi: 10.1038/s41586-021-03589-x, PMID: 34194047 PMC8439151

[B238] Yang Y. Wang T. Fu Y. Li X. Yu F. (2025). TRIM28 functions as SUMO ligase to SUMOylate TRAF6 and regulate NF-κB activation in HBV-replicating cells. Hepatol. Int 19, 529–546. doi: 10.1007/s12072-025-10779-6, PMID: 39920527

[B239] Yoneyama M. Kato H. Fujita T. (2024). Physiological functions of RIG-I-like receptors. Immunity 57, 731–751. doi: 10.1016/j.immuni.2024.03.003, PMID: 38599168

[B240] Yuan P. Yan J. Wang S. Guo Y. Xi X. Han S.. (2021). Trim28 acts as restriction factor of prototype foamy virus replication by modulating H3K9me3 marks and destabilizing the viral transactivator Tas. Retrovirology 18, 38. doi: 10.1186/s12977-021-00584-y, PMID: 34903241 PMC8670036

[B241] Zeng L. Yap K. L. Ivanov A. V. Wang X. Mujtaba S. Plotnikova O.. (2008). Structural insights into human KAP1 PHD finger–bromodomain and its role in gene silencing. Nat. Struct. Mol. Biol. 15, 626–633. doi: 10.1038/nsmb.1416, PMID: 18488044 PMC3331790

[B242] Zhai Y. Zhang M. An X. Zhang S. Kong X. Li Q.. (2021). TRIM28 maintains genome imprints and regulates development of porcine SCNT embryos. Reproduction 161, 411–424. doi: 10.1530/REP-20-0602, PMID: 33539314

[B243] Zhang Y. Wan X. Qiu L. Zhou L. Huang Q. Wei M.. (2023b). Trim28 citrullination maintains mouse embryonic stem cell pluripotency via regulating Nanog and Klf4 transcription. Sci. China Life Sci. 66, 545–562. doi: 10.1007/s11427-022-2167-3, PMID: 36100837

[B244] Zhang F.-L. Yang S.-Y. Liao L. Zhang T.-M. Zhang Y.-L. Hu S.-Y.. (2023a). Dynamic SUMOylation of MORC2 orchestrates chromatin remodelling and DNA repair in response to DNA damage and drives chemoresistance in breast cancer. Theranostics 13, 973–990. doi: 10.7150/thno.79688, PMID: 36793866 PMC9925317

[B245] Zhang H. Zheng H. Zhu J. Dong Q. Wang J. Fan H.. (2021). Ubiquitin-modified proteome of SARS-coV-2-infected host cells reveals insights into virus–host interaction and pathogenesis. J. Proteome Res. 20, 2224–2239. doi: 10.1021/acs.jproteome.0c00758, PMID: 33666082

[B246] Zhang L. Zhu C. Guo Y. Wei F. Lu J. Qin J.. (2014). Inhibition of KAP1 enhances hypoxia-induced kaposi’s sarcoma-associated herpesvirus reactivation through RBP-Jκ. J. Virol. 88, 6873–6884. doi: 10.1128/JVI.00283-14, PMID: 24696491 PMC4054365

[B247] Zhao X. (2018). SUMO-mediated regulation of nuclear functions and signaling processes. Mol. Cell 71, 409–418. doi: 10.1016/j.molcel.2018.07.027, PMID: 30075142 PMC6095470

[B248] Zhao Y. Gao Y. Guyatt G. Uyeki T. M. Liu P. Liu M.. (2024). Antivirals for post-exposure prophylaxis of influenza: a systematic review and network meta-analysis. Lancet 404, 764–772. doi: 10.1016/S0140-6736(24)01357-6, PMID: 39181596 PMC11369964

[B249] Zheng L. Pan H. Li S. Flesken-Nikitin A. Chen P.-L. Boyer T. G.. (2000). Sequence-specific transcriptional corepressor function for BRCA1 through a novel zinc finger protein, ZBRK1. Mol. Cell 6, 757–768. doi: 10.1016/S1097-2765(00)00075-7, PMID: 11090615

[B250] Zhu X. Li F. Fan B. Zhao Y. Zhou J. Wang D.. (2024). TRIM28 regulates the coagulation cascade inhibited by p72 of African swine fever virus. Vet. Res. 55, 149. doi: 10.1186/s13567-024-01407-6, PMID: 39533356 PMC11559047

[B251] Zhu Q. Xiao Y. (2024). “The immune modulatory role of TIF1 proteins,” in Ubiquitination in Immune System and Immune Response. Eds. Hu H. Fu X. (Springer Nature Singapore, Singapore), 89–99. doi: 10.1007/978-981-97-7288-9_6, PMID: 39546137

[B252] Ziegler V. Deußen M. Schumacher L. Roos W. P. Fritz G. (2020). Anticancer drug and ionizing radiation-induced DNA damage differently influences transcription activity and DDR-related stress responses of an endothelial monolayer. Biochim. Biophys. Acta (BBA) - Mol. Cell Res. 1867, 118678. doi: 10.1016/j.bbamcr.2020.118678, PMID: 32061892

